# Supramolecular Nanofibers Ameliorate Bleomycin‐Induced Pulmonary Fibrosis by Restoring Autophagy

**DOI:** 10.1002/advs.202401327

**Published:** 2024-05-09

**Authors:** Debin Zheng, Jiasen Guo, Ziyi Liang, Yueyue Jin, Yinghao Ding, Jingfei Liu, Chao Qi, Kaiwen Shi, Limin Xie, Meiqi Zhu, Ling Wang, Zhiwen Hu, Zhimou Yang, Qian Liu, Xiaoxue Li, Wen Ning, Jie Gao

**Affiliations:** ^1^ Beijing Key Laboratory of Disaster Medicine Medical Innovation Research Division of the Chinese PLA General Hospital No. 28 Fu Xing Road Beijing 100853 P. R. China; ^2^ State Key Laboratory of Medicinal Chemical Biology Key Laboratory of Bioactive Materials (Ministry of Education) College of Life Sciences Nankai International Advanced Research Institute (SHENZHEN FUTIAN) Nankai University Tianjin 300071 P. R. China; ^3^ State Key Laboratory of Medicinal Chemical Biology College of Pharmacy Nankai University Tianjin 300071 P. R. China; ^4^ Department of Urology Tianjin First Central Hospital Tianjin 300192 P. R. China

**Keywords:** autophagy, peptide nanofiber, protein‐protein interactions, pulmonary fibrosis, self‐assembly

## Abstract

Idiopathic pulmonary fibrosis (IPF) is a progressive and ultimately fatal interstitial lung disease, with limited therapeutic options available. Impaired autophagy resulting from aberrant TRB3/p62 protein‐protein interactions (PPIs) contributes to the progression of IPF. Restoration of autophagy by modulating the TRB3/p62 PPIs has rarely been reported for the treatment of IPF. Herein, peptide nanofibers are developed that specifically bind to TRB3 protein and explored their potential as a therapeutic approach for IPF. By conjugating with the self‐assembling fragment (Ac‐GFFY), a TRB3‐binding peptide motif A2 allows for the formation of nanofibers with a stable α‐helix secondary structure. The resulting peptide (Ac‐GFFY‐A2) nanofibers exhibit specific high‐affinity binding to TRB3 protein in saline buffer and better capacity of cellular uptake to A2 peptide. Furthermore, the TRB3‐targeting peptide nanofibers efficiently interfere with the aberrant TRB3/p62 PPIs in activated fibroblasts and fibrotic lung tissue of mice, thereby restoring autophagy dysfunction. The TRB3‐targeting peptide nanofibers inhibit myofibroblast differentiation, collagen production, and fibroblast migration in vitro is demonstrated, as well as bleomycin‐induced pulmonary fibrosis in vivo. This study provides a supramolecular method to modulate PPIs and highlights a promising strategy for treating IPF diseases by restoring autophagy.

## Graphical abstract

A self‐assembly peptide (Ac‐GFFY‐A2) can form nanofibers with stable α‐helix secondary structure. The  TRB3‐targeting nanofibers efficiently interfere with the aberrant TRB3/p62 PPIs in activated fibroblasts and fibrotic lung tissue of mice, thereby restoring autophay. Furthermore, the peptide nanofibers effectively inhibit myofibroblast differentiation, collagen production, and fibroblast migration in vitro, as well as bleomycin‐induced pulmonary fibrosis in vivo.

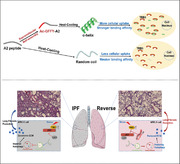

## 1 Introduction

Idiopathic pulmonary fibrosis (IPF) is a chronic, progressive even lethal interstitial lung disease characterized by abnormal accumulation of myofibroblast, deposition of extracellular matrix (ECM) around the fibroblastic foci, as well as honeycomb changes of lung parenchyma.^[^ ^1^ ^]^ IPF is the most common and severe form of pulmonary fibrosis, with a mean survival of 2–5 years.^[^ ^2^ ^]^ More worryingly, with the aggravation of environmental pollution and the increase of worldwide population aging, the incidence rate of IPF is increasing and the clinical and economic burden of IPF is sizeable.^[^ ^3^ ^]^ Clinical reports have also shown that ≈30% of coronavirus disease 2019 patients being suffered from persistent lung damage exhibit the IPF symptom after prognosis.^[^ ^4^ ^]^ Unfortunately, the pathogenesis of IPF is complicated and unclear, which limits the development of anti‐fibrotic therapies.^[^ ^5^ ^]^ Many therapies, such as clearance of senescent cells,^[^ ^6^ ^]^ mitochondria‐targeting^[^ ^7^ ^]^ and stem cell therapy, were assessed in preclinical models, and only two drugs including pirfenidone and nintedanib have been approved by the US Food and Drug administration for the IPF treatment until now.^[^ ^8^ ^]^ However, both of these drugs have significant adverse effects and only provide palliative relief.^[^ ^9^ ^]^ Therefore, it is urgent to develop a new anti‐fibrotic therapeutic strategy.

The complex pathogenesis of IPF involves multiple cell types, including the development of fibroblastic foci composed of myofibroblasts and activated fibroblasts acting as fibrotic effector cells.^[^ ^10^ ^]^ Activated fibroblast can increase the production of ECM and differentiate into myofibroblast, predominantly causing lung tissue distortion and pulmonary fibrosis.^[^ ^11^ ^]^ Furthermore, autophagy, a basic cellular homeostatic process, plays a vital regulatory role in fibroblast activation and myofibroblast differentiation.^[^ ^12^ ^]^ The activated fibroblast exhibits impaired autophagy, typically accompanied by the accumulation of p62 protein, an important selective autophagic receptor.^[^ ^13^ ^]^ The p62‐mediated autophagy dysfunction promotes fibroblast activation and myofibroblast differentiation, driving the ECM excessive deposition even the progression of pulmonary fibrosis.^[^ ^13^, ^14^ ^]^ Interestingly, recent advancements have revealed that the pseudokinase tribbles homolog 3 (TRB3), a stress and metabolic sensor, is found to be upregulated in various human diseases, including pulmonary fibrosis.^[^ ^15^ ^]^ The upregulated TRB3 protein can hinder p62 binding to LC3 and ubiquitinated substrates by interacting with p62 protein, thereby leading to its accumulation and the suppression of autophagic flux.^[^ ^16^ ^]^ Therefore, we hypothesize that interrupting the TRB3/p62 interaction to regulate autophagy may be an alternative and effective strategy to alleviate the IPF disease.

Currently, modulating protein‐protein interactions (PPIs) has emerged as a successful strategy for alleviating human diseases.^[^ ^17^ ^]^ Several PPIs modulators have progressed into clinical studies or even received regulatory approval for marketing.^[^ ^18^ ^]^ Due to its suitable molecular weight and strong protein affinity, the peptide has received expansive attention to modulate the PPIs.^[^ ^19^ ^]^ The specific secondary conformation of peptides plays a vital role in protein docking.^[^ ^20^ ^]^ Moreover, only a few of methods can be used to regulate and retain the secondary conformation of peptide, such as stapling peptide and supramolecular approach.^[^ ^21^ ^]^ In our previous study, the potential of supramolecular self‐assembly has been demonstrated in constraining the secondary conformation of peptides, allowing the formation of bioactive nanomaterials that can mimic desired proteins and bind to the target protein.^[^ ^22^ ^]^ Given that, we herein utilized the supramolecular self‐assembly method to constrain the secondary conformation of TRB3‐binding peptide fragment (A2, GGWLTRLLQTK) derived from autophagy related p62 protein, the resulting peptide nanofibers with stable conformation are capable of disturbing the aberrant TRB3/p62 PPIs to restore autophagy, eventually ameliorating bleomycin‐induced pulmonary fibrosis.

## 2 Results and Discussion

### 2.1 Abnormal TRB3/p62 PPIs Impaired Autophagic Flux in Pulmonary Fibrosis

**Figure 1:**
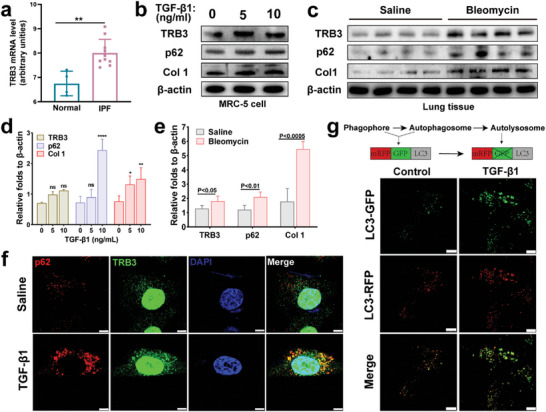
Enhanced TRB3/p62 PPIs and autophagy suppression in pulmonary fibrosis. a) TRB3 mRNA expression was upregulated in IPF fibroblasts from the GDS4580, two‐tailed Student's t‐test ^**^ *p*<0.01. b) Western bolt analysis of TRB3, p62 and Col 1 from MRC‐5 cells treated with different concentrations of TGF‐β1 for 24 h. c) Western bolt analysis for TRB3, p62, and Col 1 of lung tissue from normal mice and bleomycin‐injured mice. d) Representative immunoblots from (b) and the ratio of the indicated protein to β‐actin (n = 3, two‐way ANOVA). e) Representative immunoblots from (c) and the ratio of the indicated protein to β‐actin (n = 4, two‐way ANOVA), ^*^ *p*<0.05, ^**^ *p*<0.01, ^***^ *p*<0.001, ^****^ *p*<0.0001. f) Immunostaining images depict expression and co‐localization of the cargo protein p62 and TRB3 in MRC‐5 cells treated with 0 or 10 ng mL^−1^ TGF‐β1 for 24 h, the nucleus stained by DAPI, bar represents 7.5 µm. g) MRC‐5 cells were infected with mRFP‐GFP‐LC3 adenovirus. After 24 h, the cells were treated with 0 or 10 ng/mL TGF‐β1 for 24 h, and the autophagic flux rate was detected with Live Cell Imaging Microscopy, bar represents 10 µm.

To illustrate the relationship between TRB3/p62 PPIs and pulmonary fibrosis. We first evaluated the expression of TRB3, p62, and Collagen 1 (Col 1) in vitro. Fibroblasts are the principal cell type responsible for regulating collagen and ECM homeostasis.^[^ ^11a^ ^]^ According to the statistical data from GEO RNA‐seq database (GDS4580), the TRB3 mRNA expression of IPF fibroblasts was higher than that of normal fibroblasts (**Figure** **1**a). The transforming growth factor β1 (TGF‐β1), a profibrotic factor, could promote myofibroblast differentiation, fibroblast migration, and collagen production.^[^ ^23^ ^]^ TGF‐β1 activated MRC‐5 cell was selected as fibrosis model in vitro. We next determined the TRB3 and p62 protein expression in normal and activated MRC‐5 cells (TGF‐β1 stimulation for 24 h) by western bolt assay, respectively. As shown in Figure 1b,d, the protein expression of TRB3 and p62 were both upregulated, accompanied by elevated protein levels of Col 1 in activated MRC‐5 cells, and a positive correlation was observed between their expression and TGF‐β1 concentration. Bleomycin was used as a pulmonary fibrosis inducer in vivo.^[^ ^10a^ ^]^ Consistently, the expression of TRB3, p62, and Col 1 in fibrotic lung tissue significantly increased in comparison to normal lung tissue (Figure 1c,e). Furthermore, immunofluorescence analysis revealed that activated MRC‐5 cells exhibited elevated expression of TRB3 and accumulation of p62, as well as enhanced co‐localization of TRB3 with p62, compared with normal MRC‐5 cells (Figure 1f), indicating that enhanced TRB3/p62 PPIs occurs in MRC‐5 cells stimulated by profibrotic factor (TGF‐β1). We examined the autophagic flux by infecting mRFP‐GFP‐LC3 expressing adenovirus to MRC‐5 cells. Based on the lysosomal quenching of GFP, Figure 1g revealed that the level of autophagosome was increased in TGF‐β1 group, suggesting that the accumulation of p62 caused impaired autophagic flux in activated MRC‐5 cells. Collectively, these data revealed that the pulmonary fibrosis was associated with abnormal TRB3/p62 PPIs and the p62‐mediated autophagy suppression.

### 2.2 Peptide Nanofibers Interfere with Abnormal TRB3/p62 PPIs by Binding to TRB3 Protein

**Figure 2:**
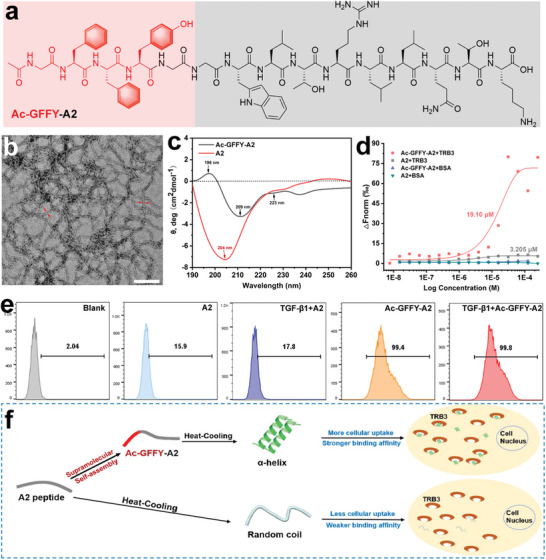
Peptide nanofibers specifically bind to the TRB3 protein. a) Chemical structure of the self‐assembly peptide Ac‐GFFY‐A2 (A2 is TRB3 targeting fragment). b) TEM images of Ac‐GFFY‐A2 (0.5 wt%) peptide nanofibers after the heat‐cooling process, bar represents 100 nm. c) The CD spectrum both of Ac‐GFFY‐A2 (0.5 wt%) and A2 (0.5 wt%) in saline buffer. d) The MST analysis of Ac‐GFFY‐A2 or A2 binding to TRB3 protein, BSA protein as negative control group. e) Flow cytometry analysis for cellular uptake of NBD labeled different peptides (100 µm) after incubation with MRC‐5 cells for 4 h. f) Schematic explaining the supramolecular self‐assembly strategy regulated the secondary conformation of A2 peptide to boost cellular uptake and protein binding affinity.

To verify our hypothesis, we utilized the self‐assembling factor (Acetyl‐Gly‐Phe‐Phe‐Tyr, Ac‐GFFY) to regulate the secondary conformation of A2 peptide (GGWLTRLLQTK) from p62 UBA domain binding with TRB3.^[^ ^16^ ^]^ The self‐assembling factor Ac‐GFFY was covalently attached to the N‐terminal of peptide A2 to introduce Ac‐GFFY‐A2 (**Figure** **2**a) as a modulator of the TRB3/p62 PPIs, non‐self‐assembling peptide A2 as the control group. Both of those peptide compounds were synthesized by standard solid phase peptide synthesis (SPPS), purified by reversed‐phase high‐performance liquid chromatography (HPLC), and identified through the high‐resolution mass spectrometry (HR‐MS, Figures S4 and S5, Supporting Information). The 0.5 wt% of peptide Ac‐GFFY‐A2 could observe an obvious “Tyndall path” in saline buffer (0.9% NaCl) after the heating‐cooling process (Figure S13, Supporting Information), compared with peptide A2 solution (0.5 wt%, saline buffer). The critical aggregation concentration (CAC) of those compounds was detected by dynamic light scattering. The CAC value of Ac‐GFFY‐A2 (48.04 µm) was approximately nine times higher than that of A2 (416.93 µm) in Figures S15 and S16 (Supporting Information). Furthermore, a transmission electron microscope (TEM) was used to observe the nanostructures formed by different compounds. Ac‐GFFY‐A2 could self‐assemble and form entangled nanofibers with a diameter of 8–12 nm (Figure 2b). However, the TEM image of A2 revealed amorphous structures in the saline buffer (Figure S17, Supporting Information). These results demonstrated that the self‐assembling motifs “Ac‐GFFY” played a vital role in initiating the peptide self‐assembly.

To determine the effects of the self‐assembly factor “Ac‐GFFY” on the secondary structure of the A2 peptide fragment, Circular dichroism (CD) and Fourier transform infrared (FTIR) measurements were performed. As depicted in Figure 2c, the CD result indicated that Ac‐GFFY‐A2 tended to form α‐helix structure with characteristics at ≈196, ≈209, and ≈223 nm, A2 adopted random coil conformations, with a strongly negative peak at ≈204 nm. Consistently, as shown in Figure S19 (Supporting Information), the FTIR data of Ac‐GFFY‐A2 exhibited a peak at ≈1655 cm^−1^, which is characteristic of α‐helix conformations,^[^ ^24^ ^]^ and that of A2 exhibited a peak at ≈1256 cm^−1^, characteristic of random coil conformations.^[^ ^25^ ^]^ We subsequently assessed the binding affinity between Ac‐GFFY‐A2 or A2 peptides to the human TRB3 protein or bovine serum albumin (BSA) using microscale thermophoresis (MST) assay, with BSA serving as the control group. The MST results (Figure 2d) showed that the Ac‐GFFY‐A2 strongly interacts with TRB3, as demonstrated by with a binding constant (K_D_) value of ≈19.10 µm. Due to the smaller thermophoresis range, A2 exhibited a weak binding to TRB3, although the K_D_ value was ≈3.21 µM.^[^ ^26^ ^]^ Moreover, both the Ac‐GFFY‐A2 and A2 displayed no binding with BSA, indicating that the binding of A2 to TRB3 was a specific interaction. Our previous study exhibited that the self‐assembly method could boost the cellular uptake level of peptides. Therefore, we labeled the peptide with the fluorescence group 7‐nitro‐1,2,3‐benzoxadiazole (NBD) to determine the cellular uptake via flow cytometer. The NBD‐labeled peptide was incubated with both of normal and activated MRC‐5 cells for 4 h, respectively. The fluorescence intensity shown in Figure 2e displayed that the cellular uptake of self‐assembling peptide (NBD‐GFFY‐A2) was more abundant than that of NBD‐A2. Collectively, under the action of supramolecular self‐assembly, the peptide assemblies with stable secondary conformation can not only be effectively absorbed by MRC‐5 cells but also have the potential to interfere with TRB3/p62 PPIs.

### 2.3 Peptide Nanofibers Restore Autophagy by Disturbing Abnormal TRB3/p62 PPIs In Vitro

**Figure 3:**
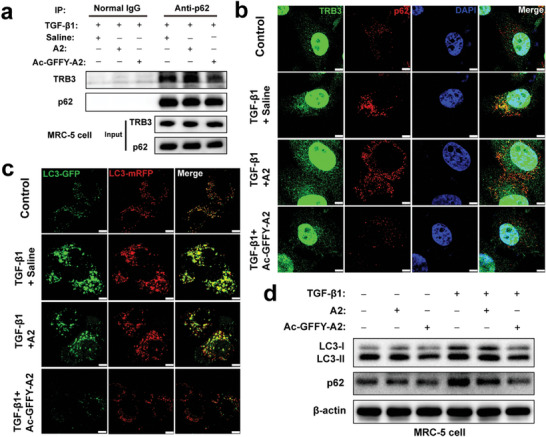
TRB3‐targeting peptide (Ac‐GFFY‐A2) nanofibers disrupt TRB3/p62 PPIs and restore autophagy. a) Co‐IP analysis for TRB3/p62 PPIs in MRC‐5 cells treated with TGF‐β1 (10 ng/mL), saline, A2 (50 µm) or Ac‐GFFY‐A2 (50 µm), respectively. + represents treated,–represents untreated. b) CLSM images depict the co‐localization of the protein p62 and TRB3 in MRC‐5 cells treated with different methods. c) After MRC‐5 cells were infected with mRFP‐GFP‐LC3 adenovirus, the cells were treated with different methods, and the autophagic flux rate was detected with Live Cell Imaging Microscopy, bar represents 10 µm. d) Western bolt analysis for autophagic marker protein LC3‐ II/LC3‐ I and p62 in MRC‐5 cells treated with TGF‐β1 (10 ng/mL), saline, A2 (50 µm) or Ac‐GFFY‐A2 (50 µm), respectively. + represents treated,–represents untreated.

The abnormal TRB3/p62 PPIs inhibit the function of p62, resulting in the suppression of autophagic flux. To acquire a non‐toxic concentration of peptide for subsequent experiments, the cytocompatibility of those two peptides for MRC‐5 cells was first detected by 3‐(4,5‐Dimethylthiazol‐2‐yl)−2,5‐diphenyltetrazolium bromide (MTT) experiment. The result in Figure S20 (Supporting Information) shows that the peptide does not have a significant growth inhibitory effect on normal cells below the concentration of 100 µm. We further investigated whether the Ac‐GFFY‐A2 nanofibers could effectively disrupt the TRB3/p62 PPIs to regulate the autophagic flux in vitro. As shown in **Figure** **3**a, the co‐immunoprecipitation (co‐IP) assay demonstrated that the Ac‐GFFY‐A2 treatment successfully inhibited the TRB3/p62 PPIs. Similarly, confocal laser scanning microscopy (CLSM) images provided visual evidence that Ac‐GFFY‐A2 impeded the co‐localization of TRB3 and p62 (Figure 3b; Fgures S21 and S22, Supporting Information), indicating the peptide nanofibers effectively disrupted the TRB3/p62 PPIs in activated MRC‐5 cells. On the contrary, the non‐self‐assembling peptide A2 exhibited minimal capacity to disrupt the TRB3/p62 PPIs. Additionally, treatment of activated MRC‐5 cells with Ac‐GFFY‐A2 resulted in the restoration of autophagic flux, as evidenced by increased LC3‐II:LC3‐I ratio and reduced levels of p62 protein, compared to A2 or saline‐treated activated MRC‐5 cells (Figure 3d; Figure S23, Supporting Information). The western bolt analysis did not show that the peptide nanofibers have ability to influence the autophagic behavior of normal MRC‐5 cells. Consistently, the results shown in Figure 3c revealed a higher abundance of ectopically expressed mRFP‐GFP‐LC3 as red and yellow speckles in the TGF‐β1 plus saline group compared to the TGF‐β1 plus Ac‐GFFY‐A2 group and the control group, suggesting that the Ac‐GFFY‐A2 peptide assemblies could restore the impaired autophagic flux in the activated MRC‐5 cells. In contrast, the CLSM picture exhibited similar results to western bolt data that the level of LC3‐GFP was increased in the TGF‐β1 plus A2 group, implying that autophagic flux was impaired and barely increased by A2 peptide. Those results indicated that the Ac‐GFFY‐A2 acted as a promising PPIs modulator and autophagy‐restored peptide via disturbing the abnormal TRB3/p62 PPIs.

### 2.4 Peptide Nanofibers Hinder the Development of Pulmonary Fibrosis by Restoring Autophagy In Vitro

**Figure 4:**
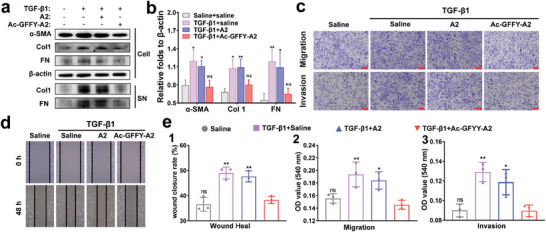
TRB3‐targeting peptide nanofibers inhibit TGF‐β1‐induced fibroblast activation, ECM production, and fibroblast migration in vitro. a) Western bolt analysis for α‐SMA, Col 1, and FN in cell extracts (Cell) and Col 1 and FN (fibronectin) in the medium supernatant (SN). + represents treated,–represents untreated. b) Representative immunoblots from (a) and the gray value ratio of the indicated protein to β‐actin (n = 3, two‐way ANOVA), ^*^ *p*<0.05, ^**^ *p*<0.01. c) Migration and invasion assay of MRC‐5 cells treated with saline, A2 (50 µm) or Ac‐GFFY‐A2 (50 µm) in the absence or presence of TGF‐β1 at 24 h, stained with 0.5% crystal violet, bar represents 50 µm. d) Wound‐healing assay of MRC‐5 cells treated with saline, A2, or Ac‐GFFY‐A2 in the absence or presence of TGF‐β1 at indicated times. e) The rate of wound closure was determined at 48 h, and the rate of migration and invasion was quantified using the OD value at 540 nm. All experiments were conducted in triplicate (n = 3, one‐way ANOVA), ^*^ *p*<0.05, ^**^ *p*<0.01.

The progression of pulmonary fibrosis is closely associated with impaired autophagic flux by promoting the differentiation of fibroblast into myofibroblast. Given that Ac‐GFFY‐A2 peptide nanofibers have ability to restore the impaired autophagy, we proceeded to investigate their potential anti‐fibrotic effect. Fibroblasts and myofibroblasts are activated after tissue injury and in IPF patients, leading to increased ECM production and enhanced fibroblast migratory capacity. As shown in **Figure** **4**a,b, the protein expression of α‐SMA (a marker of myofibroblast differentiation) and two ECM components (col 1 and fibronectin) significantly increased after MRC‐5 cells treated with TGF‐β1 but remarkably reduced by Ac‐GFFY‐A2 nanofibers intervention, while the A2 hardly inhibited fibroblast to myofibroblast differentiation and overexpression of those ECM components. In addition, only Ac‐GFFY‐A2 group exhibited a decline in the rate of migration and invasion among those groups stimulated by TGF‐β1, suggesting the inhibition of fibroblast migration and invasion by Ac‐GFFY‐A2 (Figure 4c–e). Taken together, those data revealed that the peptide nanofibers incorporating TRB3‐specific targeting motifs can effectively alleviate pulmonary fibrosis by restoring impaired autophagic flux in vitro.

### 2.5 Self‐Assembly TRB3‐Targeting Peptide Effectively Accumulate and Retain in Fibrosis Lung Tissue

**Figure 5:**
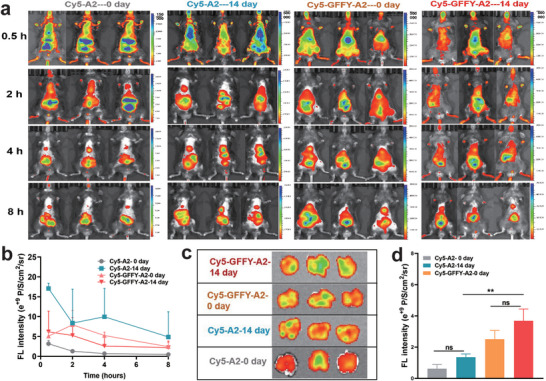
TRB3‐targeting peptide selectively accumulates in the lung tissue. a) NIR fluorescence imaging of the bio‐distribution of sulfo‐Cy5 labeled peptide conjugates (*i.v*., 0.3 mg per mouse) in the wild male C57BL/6 mice (0 day) and 2U kg^−1^ bleomycin‐treated male C57BL/6 mice (14 day) in vivo. b) Quantitative fluorescence analyses of (a) at the corresponding time in the lung area of mice. c) NIR fluorescence images of excised lung tissue post injection at 8 h. d) Quantitative fluorescence analyses of (c) for corresponding tissue ex vivo. One‐way ANOVA, mean± SD, ^**^ *p* < 0.01, ns represent no significance.

The efficient accumulation and retention of peptides at the lesion site is an important prerequisite for the therapeutic effect. Therefore, the accumulation and retention capacity of the peptide was investigated by changing the Acetyl group to sulfo‐cyanine‐5, enabling the new synthetic molecules (entitled Cy5‐GFFY‐A2 and Cy5‐A2, Figures S9–S11, Supporting Information) to be visualized through in vivo imaging systems. The pulmonary fibrosis model was established in male C57BL/6 mice by intratracheal administration of bleomycin at a dose of 2 U kg^−1^ for 14 days (namely 14 day), while the wild mice as a control group (namely 0 day). The mice were administrated by sulfo‐Cy5 labeled peptide (0.3 mg per mouse) in intravenous injection. As shown in **Figure** **5**a,b, the sulfo‐Cy5 labeled peptide was rapidly enriched at the lung tissue during 0.5–2 h after administration, with the largest fluorescence intensity. The quantitative fluorescence statistics in the lung area showed that Cy5‐GFFY‐A2 has better lung tissue retention capacity and slower degradation rate than that of the Cy5‐A2 group. To eliminate fluorescence errors caused by differences in epidermal‐to‐lung tissue depths among mice, the main organs were harvested 8 h after administration to further investigate the biodistribution of peptide in the body of the mouse ex vivo. As depicted in Figure 5c,d, the fluorescence intensity of Cy5‐GFFY‐A2 group was significantly higher than that of the Cy5‐A2 group, which was consistent with the in vivo image result, indicating that self‐assembly factor “GFFY” was beneficial to improve the accumulation and retention of A2 peptide fragment in the lung tissue. Furthermore, the fluorescence intensity of those two pulmonary fibrosis model groups (14 day) was increased compared to the normal group (0 day) when the same peptide was injected, which might be attributed to the enhanced expression level of TRB3 protein in fibrosis lung tissue. In addition, the fluorescence intensity of the lung tissue was higher than that of other organs other than the liver tissue. And the ex vivo images exhibited a strong fluorescence intensity in the liver (Figure S24, Supporting Information), indicating that the peptide was mainly removed by hepatic clearance. Together, these results showed that the TRB3‐targeting peptide was capable of effectively accumulating and retaining in fibrosis lung tissue, which was essential for ameliorating the pulmonary fibrosis.

### 2.6 Peptide Nanofibers have a Potent Anti‐Fibrotic Activity In Vivo

**Figure 6:**
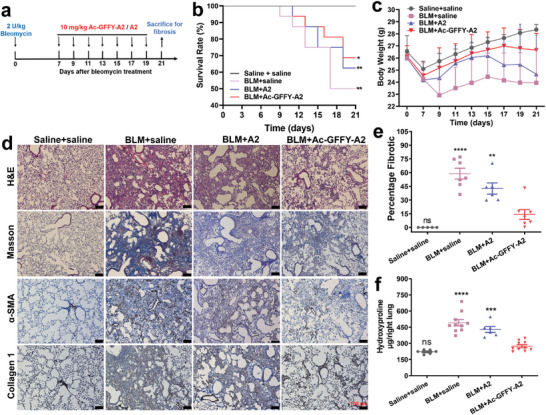
TRB3‐targeting peptide nanofibers ameliorate bleomycin‐induced pulmonary fibrosis in mice. a) Schematic representation of the therapeutic timeline of Ac‐GFFY‐A2 (10 mg/kg) or A2 (no‐self‐assembling peptide) to mice with established fibrosis model following bleomycin‐induced lung injury for 7 days, mice intratracheally injected with saline were used as a sham control, therapeutic formulation administrated with *i.v*. injection. b) Percentages of survived mice during a 21‐day period, (n = 16, Log‐rank (Mental‐Cox) test). c) The curve of average mice body weight, (saline + saline group: n = 6, BLM + saline group: n = 12, BLM + A2 group: n = 8, BLM + Ac‐GFYY‐A2 group: n = 12). d) Representative images of lung sections stained with hematoxylin and eosin (H&E), Masson's trichrome as well as immunohistochemical analysis for α‐SMA and Collagen 1, bar represents 100 µm. e) Lung fibrotic score analysis shows percentages of fibrotic areas in lung sections, one‐way ANOVA. f Hydroxyproline contents in lung tissue. One‐way ANOVA, ^****^ *p*<0.0001, ^***^ *p*<0.001, ^**^ *p*<0.01.

We further evaluated the anti‐fibrotic effect of Ac‐GFFY‐A2 peptide nanofibers in vivo. As shown in Figure 5a, the C57BL/6 mice pulmonary fibrosis model was established by intratracheal administration of bleomycin at a dose of 2 U kg^−1^ and the mice 7 days later were treated with intravenous (*i.v*.) injections of either saline (sham control group), A2, or Ac‐GFFY‐A2. As expected, the group of mice treated with Ac‐GFFY‐A2 demonstrated a higher survival rate of 68.75% (**Figure** **6**b), compared to the mice treated with saline (50.00%) or A2 (62.50%). Consistently, the group of Ac‐GFFY‐A2 (red line in Figure 6c) exhibited a partial recovery from the body weight loss induced by lung fibrosis, suggesting that Ac‐GFFY‐A2 has a positive impact on the therapy of pulmonary fibrosis in mouse model. Importantly, the severity of bleomycin‐induced pulmonary fibrosis was evaluated by hematoxylin‐eosin (H&E) staining in each group, and optical images in Figure 6d and Figure S25 (Supporting Information) shows that Ac‐GFFY‐A2 treatment could mitigate the lung fibrosis. Quantification of fibrotic left lung sections clearly illustrated a significant decrease in fibrosis following Ac‐GFFY‐A2 treatment, as compared to the administration of saline or A2 (Figure 6e), which was further supported by decreased protein levels of Col 1 and α‐SMA (Figure 6d). Additionally, the mice treated with Ac‐GFFY‐A2 exhibit decreased levels of collagen accumulation, as evidenced by the Masson trichrome staining (Figure 6d). Consistent with the histological analysis, the bleomycin challenge resulted in a marked increase in the hydroxyproline content up to ≈480 µg per right lung tissue, which decreased to ≈280 µg per right lung tissue by Ac‐GFFY‐A2 treatment, nearly back to the level of sham control group (Figure 6f). The observed reductions in fibrosis, collagen accumulation, and protein levels of Col 1 and α‐SMA strongly support the efficacy of Ac‐GFFY‐A2 in combating fibrotic processes, confirming that the peptide nanofibers as a TRB3/p62 PPIs modulator with a potent anti‐fibrotic activity.

### 2.7 Peptide Nanofibers Ameliorate Bleomycin‐Induced Pulmonary Fibrosis by Restore Autophagy

**Figure 7:**
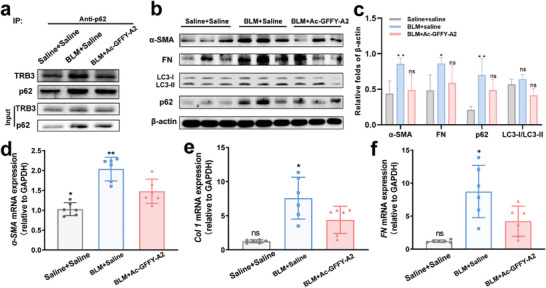
Disrupting the TRB3/p62 PPIs restores the autophagy and inhibits the progression of pulmonary fibrosis. a) Co‐IP analysis for TRB3/p62 interaction in the lung tissues. b) Western bolt analysis for the α‐SMA, FN, LC3‐II/LC3‐I, and p62 in the lung tissues. c) Grayscale analysis of the ratio of the indicated protein to β‐actin in lung tissue, (n = 3, two‐way ANOVA). The quantitative polymerase chain reaction (q‐PCR) analysis for mRNA level in lung tissues of d) α‐SMA, e) Col 1, f) FN, one‐way ANOVA, ^**^ *p*<0.01, ^*^ *p*<0.05, ns represents no significance.

We attempted to elucidate the mechanism of Ac‐GFFY‐A2 peptide nanofibers in ameliorating bleomycin‐induced lung fibrosis at the molecular level. The nanofibers were administered to the lung fibrosis mice model via intravenous (*i.v*.) injection, saline treatment as the control group. The mice were sacrificed on day 21 and lung tissues were harvested for the following detection. As shown in **Figure** **7**a, the TRB3/p62 PPIs in vivo were detected by co‐IP experiments, the co‐IP results revealed that BLM treatment (BLM + Saline group) could cause enhanced TRB3/p62 PPIs compared with the saline + saline group, while the TRB3‐targeting nanofibers could disturb the interaction between TRB3 and p62 in lung tissue, which caused a reduction in accumulation of p62 and an increase in the ratio of LC3‐II/LC3‐I (Figure 7b,c). Those results indicated that the nanofibers possessed the ability to restore impaired autophagic flux in vivo. Meanwhile, the results of q‐PCR exhibited that mRNA expression levels of Col 1, FN, and α‐SMA in lung tissues were significantly lower than those in the saline treatment control group (Figure 7d–f), which is consistent with the western bolt results. Those results confirmed the potential therapeutic efficacy of peptide nanofibers in pulmonary fibrosis at both the transcriptional and translational levels. Taken together, those data demonstrated that the peptide nanofibers as a PPIs modulator can ameliorate bleomycin‐induced pulmonary fibrosis by restoring impaired autophagy.

**Scheme 1:**
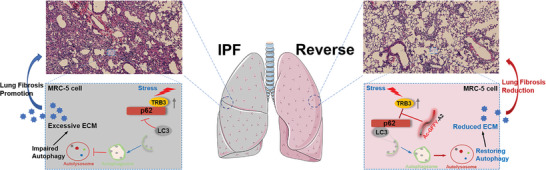
Proposed mechanism of the autophagy‐associated lung fibrosis progression or the Ac‐GFFY‐A2 peptide nanofibers against lung fibrosis in vivo.

The pathogenesis of IPF is not completely understood and needs to be further expounded, but IPF progression is closely related to the fibroblast‐to‐myofibroblast differentiation and the excessive deposition of ECM proteins, resulting to lung tissue distortion and even death.^[^ ^8^ ^]^ Recent evidence suggests that the insufficient autophagy is linked to cellular senescence, which has been proposed an important factor for IPF and other fibrotic diseases.^[^ ^27^ ^]^ Impaired autophagy can cause the acceleration of fibroblast senescence, myofibroblast differentiation, and ECM production.^[^ ^28^ ^]^ Here, this study developed a potential peptide‐based treatment for IPF by restoring autophagy (**Scheme** **1**), while earlier studies have explored the effect of antioxidants, mTOR inhibitors, and et.al on IPF treatment by regulating autophagy.^[^ ^8^ ^]^

The impaired autophagy caused by the profibrotic factor TGF‐β1 has been found to evoke fibroblast to myofibroblast differentiation, which is a critical step in the generation of pulmonary fibrosis.^[^ ^13^ ^]^ Our study verified that lung fibroblasts stimulated by the TGF‐β1 exhibited an accumulation of p62 protein and impaired autophagic flux compared to normal fibroblasts. Furthermore, impaired autophagic flux is accompanied by aberrant TRB3/p62 PPIs in a variety of disease models, such as cancer and hepatic fibrosis.^[^ ^15^, ^16^ ^]^ Generally, overexpressed TRB3 protein interacted with p62 protein and hindered its binding to MAP1LC3/LC3, thereby obstructing autophagic flux. We also confirmed upregulation of TRB3 protein level and enhanced TRB3/p62 PPIs in activated fibroblasts and BLM‐induced mouse models of pulmonary fibrosis. Moreover, when overexpressed TRB3 protein in activated fibroblast and BLM‐injured mice was competitively bound by TRB3‐targeting peptide nanofibers, the binding of p62 protein to TRB3 was reduced, and the autophagic flux was also restored, with the expression of α‐SMAand two ECM components (col 1 and fibronectin) decreased consequently. Those data indirectly suggest TRB3‐associated autophagy dysfunction in pulmonary fibrosis. However, we have yet not to investigate the underlying molecular mechanisms between TRB3 and autophagic flux in pulmonary fibrosis but only focused on exploring whether interference with TRB3/p62 PPIs can regulate autophagy flux, thereby mitigating pulmonary fibrosis progression.

Peptides have demonstrated efficacy in the regulation of PPIs, and peptide fragments exhibiting a stable secondary structure are imperative for protein binding.^[^ ^29^ ^]^ In our study, the self‐assembling peptide Ac‐GFFY‐A2 can form nanofibers with stable secondary structures through non‐covalent bond forces. These MST and co‐IP results demonstrate that the peptide nanofibers could competitively bind to TRB3 protein and interfere with the TRB3/p62 PPIs. However, the synthetic peptides from native proteins generally adopt random coil conformations to keep thermal dynamic equilibrium,^[^ ^22a^ ^]^ so the peptide A2 hardly maintains the bioactive conformations of native proteins to bind TRB3. Consistently, the non‐self‐assembling peptide A2 exhibited weak binding affinity toward TRB3 and displayed minimal cellular uptake capacity, resulting in its inability to effectively interfere with the TRB3/p62 interaction in vitro and in vivo. Therefore, supramolecular self‐assembly could be an efficient and convenient tool for designing peptide‐based PPIs modulators. On the other hand, TRB3, as a stress sensor, exhibits interactions not only with p62 but also with several other vital signaling factors like AKT, NFκB, and MAPK, enabling it to regulate numerous biological functions.^[^ ^15b^ ^]^ In activated fibroblasts, the overexpression of the TRB3 protein may dysregulate the function of the aforementioned proteins, leading to abnormal effects. Therefore, Ac‐GFFY‐A2 nanofibers have the potential to modulate the TRB3/p62 PPIs and exert effects at other sites, which merit further investigation.

## 3 Conclusion

In conclusion, we have utilized a supramolecular self‐assembly strategy to successfully construct a peptide‐based PPIs modulator. By combining self‐assembling peptide motifs Ac‐GFFY with TRB3‐targeting peptide motifs A2, the resulting peptide Ac‐GFFY‐A2 could form nanofibers with stable  α‐helix secondary structure after heating‐cooling process in saline buffer. We demonstrated that the nanofibers were capable of restoring impaired autophagic flux via specifically disturbing the aberrant TRB3/p62 PPIs in activated fibroblasts and fibrosis lung tissue of mice. Overall, the nanofibers exhibited the ability to inhibit myofibroblast differentiation, collagen production, and fibroblast migration in vitro, as well as bleomycin‐induced pulmonary fibrosis in vivo. Our study thus highlights a promising strategy for the treatment of pulmonary fibrosis diseases.

## 4 Experimental Section

### Materials

2‐Cl‐trityl chloride resin (1.1 mmol g^−1^) was obtain from Nankai HECHENG Co., Ltd (Tianjin). Fmoc‐amino acids and o‐benzotriazol‐1‐yl‐N,N,N’,N’‐tetramehtyluronium hexafluorophosphate (HBTU) were bought from GL Biochem (Shanghai). Chemical reagents and solvents were used as received from commercial sources. Commercially available reagents were used without further purification unless noted otherwise. Copper mesh coated with carbon was from Beijing Zhongjingkeyi Technology Co., Ltd. Recombinant human TGF‐β1 (7754‐BH) was purchased from R&D Systems. Recombinant human TRB3 (10731‐H09B) was purchased from Sino Biological. TRB3 antibody (PA5‐29887) was purchased from invitrogen, p62 antibody (66184‐1) was purchased from Proteintech, β‐actin antibody (AC026) was purchased from ABclonal Technology; Collagen I antibody (ab21286) and Fibronectin antibody (ab2413) were purchased from Abcam. α‐SMA (sc‐32251) was purchased from Santa Cruz. LC3B antibody (L7543) was purchased from Sigma‐Aldrich. Bleomycin (Blenoxane) was purchased from Nippon Kayaku Co., Ltd. AV‐CMC‐TagRFP‐SEP‐LC3‐SV40 PA was purchased from Sango Biotect (Shanghai) Co., Ltd. The human lung fibroblast MRC‐5 cells were purchased from Hunan Fenghui Biotechnology (Hunan, China), RIPM 1640 medium and penicillin/streptomycin were purchased from Gibco Corporation. Eight‐week‐old male C57BL/6 wild‐type mice were purchased from Vital River Laboratories (Beijing, China).

### General Methods

HR‐MS (Varian QFT‐ESI) was used to characterize the molecular weight of compounds. TEM (JEM100CXII) was performed at the Tecnai G2 F20 system, operating at 100 kV. Circular dichroism (CD) spectrum was obtained by a BioLogic (MOS‐450) system. Dynamic light scattering was measured by a ZETAPALS/BI‐200SM (BROOKHAVEN) system. Confocal laser scanning microscopy (CLSM) was measured by Leica TCS SP‐5. Flow cytometry (BD, FACS) was used to detect the cellular uptake. The microthermophoresis instrument (MST Monolith NT.115) was used to measure the KD values of protein‐peptide binding constant. Mice were housed in a pathogen‐free animal facility at Nankai University and all animal experiments in this work were carried out under the guidelines set by Tianjin Committee of Use and Care of Laboratory Animals, and the overall project protocols were approved by the Animal Experiments Ethical Committee of Nankai University and complied with the Guide for Care and Use of Laboratory Animals (Approval number 2022‐SYDWLL‐000418).

### Preparation of Peptides

All peptides were prepared by standard solid phase peptide synthesis (SPPS) by using 2‐chlorotrityl chloride resin and the corresponding N‐Fmoc protected amino acids with side chains properly protected. First, the C‐terminal of the first amino acid was conjugated on the resin. Anhydrous N,N’‐dimethyl formamide (DMF) containing 20% piperidine was used to remove Fmoc group. O‐Benzotriazol‐1‐yl‐N,N,N’,N’‐tetramethyluronium hexafluorophosphate (HBTU) was used as a coupling reagent, DIPEA was used as a base regent. Acetic anhydride or β‐alanine substituted NBD was used at the final step as a capping group. Lastly, the chemical cleavage liquid containing 95% TFA 2.5% H_2_O, and 2.5% Tis was used to cleave peptides derivative from resin and the mixture was concentrated by rotary evaporation. Cold diethyl ether was poured into concentrates to make the crude peptide precipitation. The precipitate was centrifuged for 7 min at a speed of 5000 rpm. Discarding the supernatant to collect solid peptide. The solid was dried by vacuum pump and then purified by HPLC to obtain the pure peptide compounds.

### Cell Line Culture

The human lung fibroblast MRC‐5 cells were maintained in Eagle's Minimum Essential Medium (MEM) with 10% fetal bovine serum (FBS), 100 U mL^−1^ penicillin, and 100 µg mL^−1^ streptomycin in 5% CO_2_ at 37 °C in a humidified atmosphere.

### Stock Solution Preparation

The Ac‐GFFY‐A2 (9.15 mg) was dissolved in saline buffer (1 mL), and an appropriate amount of Na_2_CO_3_ solution (1 m) was added to adjust the pH to 7.4. Then the solution was treated by heat‐cooling to obtain the stock solution of peptide nanofibers.

### Cytocompatibility Study

MRC‐5 cells were used to perform the cytocompatibility experiment for the Ac‐GFFY‐A2 and A2 peptides. MRC‐5 cells were seeded in 96‐well plates at a density of 4 × 10^3^ cells per well for 16 h, followed by culture medium removal and subsequent addition of the culture medium containing Ac‐GFFY‐A2 or A2 peptide. The initial concentration of all compounds was 100 µm. After 48 h, 10 µL of the MTT solution (5 mg mL^−1^) was added to each well and incubated at 37 °C for another 4 h. The medium was removed, and 100 µL of DMSO was then added to stop the reduction reaction and dissolve the purple formazan formed within the cells. The optical density of the solution was measured at 490 nm using a microplate reader (Bio RADiMark, USA).

### RNA Isolation and qRT‐PCR Analysis

Total RNA was isolated from lung tissues using TRIGene (GenStar, Beijing, China) and then reverse‐transcribed by Star ScriptII First‐strand cDNA Synthesis Mix (GenStar, Beijing, China) according to the manufacturer's instructions. qRT‐PCR was performed using qPCR SYBR Green Master Mix (Yeasen, Shanghai, China). Gene expression was determined relative to that of the endogenous reference gene (Gapdh) using the 2^−ΔΔCt^ method. The mouse primers used in this study were as follows: Acta2 [encoding α‐SMA], 5′‐GCTGGTGATGATGCTCCCA‐3′ and 5′‐GCCCATTCCAACCATTACTCC‐3′; Col1a1 [encoding type I collagen (Col1)], 5′‐CCAAGAAGACATCCCTGAAGTCA‐3′ and 5′‐TGCACGTCATCGCACACA‐3′; Fn (encoding fibronectin), 5′‐GTGTAGCACAACTTCCAATTACGAA‐3′ and 5′‐GGAATTTCCGCCTCGAGTCT‐3′; Gapdh (encoding Gapdh), 5′‐AGGTCGGTGTGAACGGATTTG‐3′ and 5′‐TGTAGACCATGTAGTTGAGGTCA‐3′.

### Live‐Cell Imaging for Autophagic Flux

According to the manufacturer's instructions, MRC‐5 cells were infected with mRFP‐GFP‐LC3 adenoviral particles. After infection, the cells were cultured for another 24 h and seeded on a CLSM dish at a density of 5 × 10^4^ cells. After adherence, the cells were treated with 0 or 10 ng mL^−1^ TGF‐β1 for 24 h. On the other hand, in order to detect whether peptide could regulate the autophagic flux, the stable infected MRC‐5 cells were seeded on a CLSM dish at a density of 5 × 10^4^ cells. After adherence, the cells were treated with saline, Ac‐GFFY‐A2 (50 µm) or A2 (50 µm) plus TGF‐β1 (10 ng mL^−1^) for 24 h. The cells solely treated by saline as control group. Discarding the medium and washing the cells with cold PBS buffer. The autophagic flux was detected by Live Cell Imaging Microscopy (Leica TCS SP5).

### Immunofluorescent Analysis

The MRC‐5 cells were seeded on the CLSM dish at a density of 6 × 10^4^ cells. After adherence, the solution containing TGF‐β1 (10 ng mL^−1^), saline plus TGF‐β1 (10 ng mL^−1^), A2 (50 µm) plus TGF‐β1 (10 ng mL^−1^), Ac‐GFFY‐A2 (50 µm) TGF‐β1 (10 ng mL^−1^), was added to incubate cells for 24 h, respectively. Then, the medium was removed and the cells were washed with cold PBS three times. The cells were fixed with 4% of Paraformaldehyde for 10 min at room temperature. Then, the solution was sucked away, and washed by cold PBS three times for a min each time. 0.1% Triton X‐100 solution was used to disrupt the cell membrane structure. Then 5% of goat serum in TBST was used to block non‐specific protein binding site and incubate cells for 1 h. Discard the blocking solution and repeat the washing step mentioned above three times. TRB3 rabbit antibody (1:100) and p62 mouse antibody (1:100) in 5% goat serum of PBS buffer were used to incubate the cells overnight at 4 °C. Washing the cells with TBST three times for 10 min each time, the Alexa Fluor647 goat anti‐mouse antibody and Alexa Fluor488 goat anti‐rabbit antibody in 5% goat serum of PBS buffer was used to incubate the cells for 1 h at room temperature. Solution of primary antibody was removed and cells were washed three times by PBS buffer and then stained with 0.5 µg mL^−1^ of DAPI for 5 min at room temperature. Operations need to be protected from light. All images were taken by a laser scanning confocal microscopy (Leica TSC SP5) at the same voltage.

### Cell Migration and Invasion Assay

For wound healing, cell monolayers were scratched with pipette tip. Cells were incubated with or without 10 ng ml^−1^ recombinant human TGF‐β1. Ac‐GFFY‐A2 (50 µm) or A2 (50 µm) was added to the medium 1 h behind to TGF‐β1 stimulation. Images were captured at 0 and 48 h after scratching, and the lesion area was measured with Image J software. Cell invasion assay was performed using transwell chambers (8‐µm pore size, Millipore). Chambers were pre‐coated with Matrigel (30 µg per well, BD). Cells were seeded into the chamber with MEM supplemented with 0.1% FBS, and the lower well was added MEM supplemented with 20% FBS. After incubated for 24 h, non‐invaded cells on the upside were removed with cotton swap, and invaded cells were fixed with 4% paraformaldehyde, stained with 0.5% crystal violet, and then photographed under a microscope. The crystal violet stain was eluted in alcohol and quantified at OD540 nm. The transwell migration assay procedure was same as invasion assay except that the chamber was not coated with matrixgel.

### Bleomycin Model of Pulmonary Fibrosis

C57BL/6 mice were intratracheally administered with bleomycin at a dose of 2 U kg^−1^ body weight as previously described.^[^ ^11a^ ^]^

### Living Image

The fibrosis mice were randomly after 14 days of bleomycin treatment, and the wild mice were so (0 day). The mice were divided into four groups (n = 3), including of Cy5‐GFFY‐A2 −14 days, Cy5‐A2‐14 days, Cy5‐GFFY‐A2‐0 days, and Cy5‐A2‐0 days. The mice were then intravenously injected with 200 µL of sulfo‐Cy5 labeled peptide (0.3 mg per mouse). Fluorescence imaging was performed using a IVIS Lumina II imaging (PerkinElmer, USA) at different time point post injection. Mice were sacrificed after 8 h post‐injection for fluorescence imaging of the major organs (i.e., heart, liver, spleen, lung, and kidney).

### Hydroxyproline Assay

Hydroxyproline content of the right lung of each mouse was measured using a conventional hydroxyproline method as previously described.^[^ ^30^ ^]^

### Histology and Immunohistochemistry

The left lungs cleared of blood were inflated with 10% neutral buffered formalin and fixed overnight. Then the tissues were embedded in paraffin and sectioned. The 5‐µm sections were stained with H&E and Masson trichrome according to the manufacturer`s instructions (Sigma–Aldrich). Immunohistochemistry was performed as previously described.^[^ ^30^ ^]^ The antibodies specific for α‐SMA and Collagen I were used for staining.

### Statistical Analysis

All results were represented as the means ± SEM. Two group comparisons were analyzed by Student's t‐test as appropriate, while multiple comparisons among three or more groups were performed using one‐way ANOVA or two‐way ANOVA, respectively; *p* ^*^ < 0.05 or *p* ^**^ < 0.01, were considered statistically significant. All analyses were performed using GraphPad Prism 8.0 or Origin 2018 software.

## Conflict of Interest

The authors declare no conflict of interest.

## Author Contributions

D.B.Z. and J.S.G. contributed equally to this work. L.W., Z.M.Y., J.G., and W.N. devised the project and supervised the research. D.B.Z. and J.S.G. performed all experiments and analyzed the data. J.F.L., Y.Y.J., C.Q., Y.H.D., L.M.X., K.W.S., M.Q.Z., Z.W.H., Z.Y.L., and Y.H.D. participated in part of the experiments. D.B.Z., X.X.L., Q.L., J.G., W.N., and Z.M.Y. wrote and revised the paper. All the authors discussed the results and have the approval of the submission of the final version of the manuscript.

## Supporting information

Supporting Information

## Data Availability

Research data are not shared.
