# Supplementary material for: Supramolecular Nanofibers Ameliorate Bleomycin‐Induced Pulmonary Fibrosis by Restoring Autophagy
Source: Adv Sci (Weinh). 2024 May 9;11(28):2401327. doi: 10.1002/advs.202401327 (PMC11267363; doi:10.1002/advs.202401327)
Supplement: Supplementary file 1 — Supporting Information [file ADVS-11-2401327-s001.pdf]

ADVANCED SCIENCE
Open Access

Supporting Information

for *Adv. Sci.*, DOI 10.1002/advs.202401327

Supramolecular Nanofibers Ameliorate Bleomycin-Induced Pulmonary Fibrosis by Restoring Autophagy

Debin Zheng, Jiasen Guo, Ziyi Liang, Yueyue Jin, Yinghao Ding, Jingfei Liu, Chao Qi, Kaiwen Shi, Limin Xie, Meiqi Zhu, Ling Wang, Zhiwen Hu, Zhimou Yang, Qian Liu, Xiaoxue Li, Wen Ning* and Jie Gao**

Supporting Information

Supramolecular Nanofibers Ameliorate Bleomycin-Induced Pulmonary Fibrosis by Restoring Autophagy

Debin Zheng[1#], Jiasen Guo[2#], Ziyi Liang[2], Yueyue Jin[2], Yinghao Ding[2], Jingfei Liu[2], Chao Qi[2], Kaiwen Shi[1], Limin Xie[2], Meiqi Zhu[1], Ling Wang[3], Zhiwen Hu[2], Zhimou Yang[2], Qian Liu[4], Xiaoxue Li[1*], Wen Ning[2*] and Jie Gao[2*]

[1]Medical Innovation Research Division of the Chinese PLA General Hospital, Beijing Key Laboratory of Disaster Medicine, No. 28 Fu Xing Road, Beijing, 100853, P. R. China

[2]Key Laboratory of Bioactive Materials, Ministry of Education, State Key Laboratory of Medicinal Chemical Biology, College of Life Sciences, Nankai University, Tianjin 300071, Nankai International Advanced Research Institute (SHENZHEN ▪ FUTIAN), P. R. China

[3]State Key Laboratory of Medicinal Chemical Biology, College of Pharmacy, Nankai University, Tianjin 300071, P. R. China

[4]Department of Urology, Tianjin First Central Hospital, Tianjin 300192, China

[#]These authors contributed equally to this work.

*Corresponding authors. Email: lixiaoxue@301hospital.com.cn (X. X. Li), ningwen108@nankai.edu.cn (W. Ning.) and chemgaojie@nankai.edu.cn (J. Gao.)

Materials: 2-Cl-trityl chloride resin (1.1 mmol/g) was obtain from Nankai HECHENG Co., Ltd (Tianjin). Fmoc-amino acids and o-benzotriazol-1-yl-N,N,N’,N’-tetramehtyluronium hexafluorophosphate (HBTU) were bought from GL Biochem (Shanghai). Chemical regents and solvents were used as received from commercial sources. Commercially available reagents were used without further purification, unless noted otherwise. Copper mesh coated with carbon was from Beijing Zhongjingkeyi Technology Co., Ltd. Recombinant human TGF-β1 (7754-BH) was purchased from R&D Systems. Recombinant human TRB3 (10731-H09B) was purchased from Sino Biological. TRB3 antibody (PA5-29887) was purchased from invitrogen, p62 antibody (66184-1) was purchased from Proteintech, β-actin antibody (AC026) was purchased from ABclonal Technology; Collagen I antibody (ab21286) and Fibronectin antibody (ab2413) were purchased from Abcam. α-SMA (sc-32251) was purchased from Santa Cruz. LC3B antibody (L7543) was purchased from Sigma-Aldrich. Bleomycin (Blenoxane) was purchased from Nippon Kayaku Co., Ltd. AV-CMC-TagRFP-SEP-LC3-SV40 PA was purchased from Sango Biotect (Shanghai) Co., Ltd. The human lung fibroblast MRC-5 cells were purchased from Hunan Fenghui Biotechnology (Hunan, China), RIPM 1640 medium and penicillin/streptomycin were purchased from Gibco Corporation. Eight-week-old male C57BL/6 wild-type mice were purchased from Vital River Laboratories (Beijing, China).

General methods: HR-MS (Varian QFT-ESI) was used to characterize the molecular weight of compounds. TEM (JEM100CXII) was performed at the Tecnai G2 F20 system, operating at 100 kV. Circular dichroism (CD) spectrum was obtained by a BioLogic (MOS-450) system. Dynamic light scattering was measured by a ZETAPALS/BI-200SM (BROOKHAVEN) system. Confocal laser scanning microscopy (CLSM) was measured by Leica TCS SP-5. Flow cytometry (BD, FACS) was used to detect the cellular uptake. The microthermophoresis instrument (MST Monolith NT.115) was used to measure the K_D values of protein-peptide binding constant. Mice were housed in a pathogen-free animal facility at Nankai University

and all animal experiments in this work were carried out under the guidelines set by Tianjin Committee of Use and Care of Laboratory Animals, and the overall project protocols were approved by the Animal Experiments Ethical Committee of Nankai University and complied with the Guide for Care and Use of Laboratory Animals (Approval number 2022-SYDWLL-000418).

Preparation of peptides: All peptides were prepared by standard solid phase peptide synthesis (SPPS) by using 2-chlorotrityl chloride resin and the corresponding N-Fmoc protected amino acids with side chains properly protected. Firstly, the C-terminal of the first amino acid was conjugated on the resin. Anhydrous N,N'-dimethyl formamide (DMF) containing 20% piperidine was used to remove Fmoc group. O-Benzotriazol-1-yl-N,N,N',N'-tetramethyluronium hexafluorophospha -te (HBTU) was used as coupling reagent, DIPEA was used as base regent. Acetic anhydride or β-alanine substituted NBD was used at the final step as capping group. Lastly, the chemical cleavage liquid containing 95% TFA 2.5% H_2O and 2.5% Tis was used to cleave peptides derivative from resin and the mixture was concentrated by rotary evaporation. Cold diethyl ether was poured into concentrates to make the crude peptide precipitation. The precipitate was centrifuged for 7 min at a speed of 5000 rpm. Discarding the supernatant to collect solid peptide. The solid was dried by vacuum pump and then purified by HPLC to obtain the pure peptide compounds.

Table S1. HPLC elution program

Time (min)	Buffer A (%) [a)]	Buffer B (%) [b)]
0.00	98.0	2.00
2.50	95.0	5.00
5.00	90.0	10.0
22.00	5.0	95.0
25.00	0.0	100.0
26.00	98.0	2.00
30.00	98.0	2.00

a) Buffer A consist of 99.9% H_2O and 0.1% TFA, b) Buffer B consist of 99.9% CH_3CN and 0.1% TFA

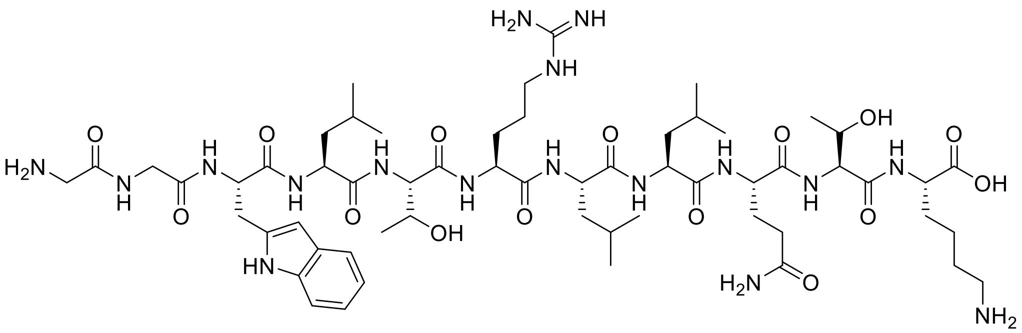

Figure S1. Chemical structure of peptide GGWLTRLLQTK (A2)

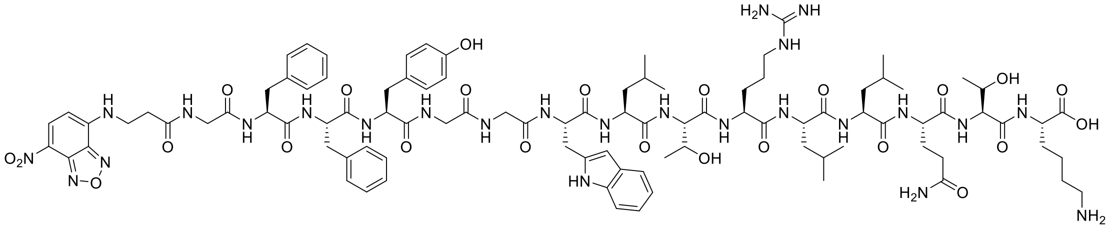

Figure S2. Chemical structure of peptide NBD-GFFY-A2

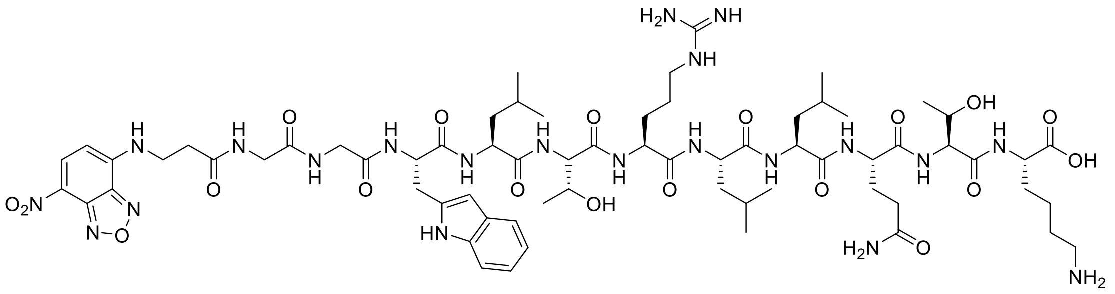

Figure S3. Chemical structure of peptide NBD- A2

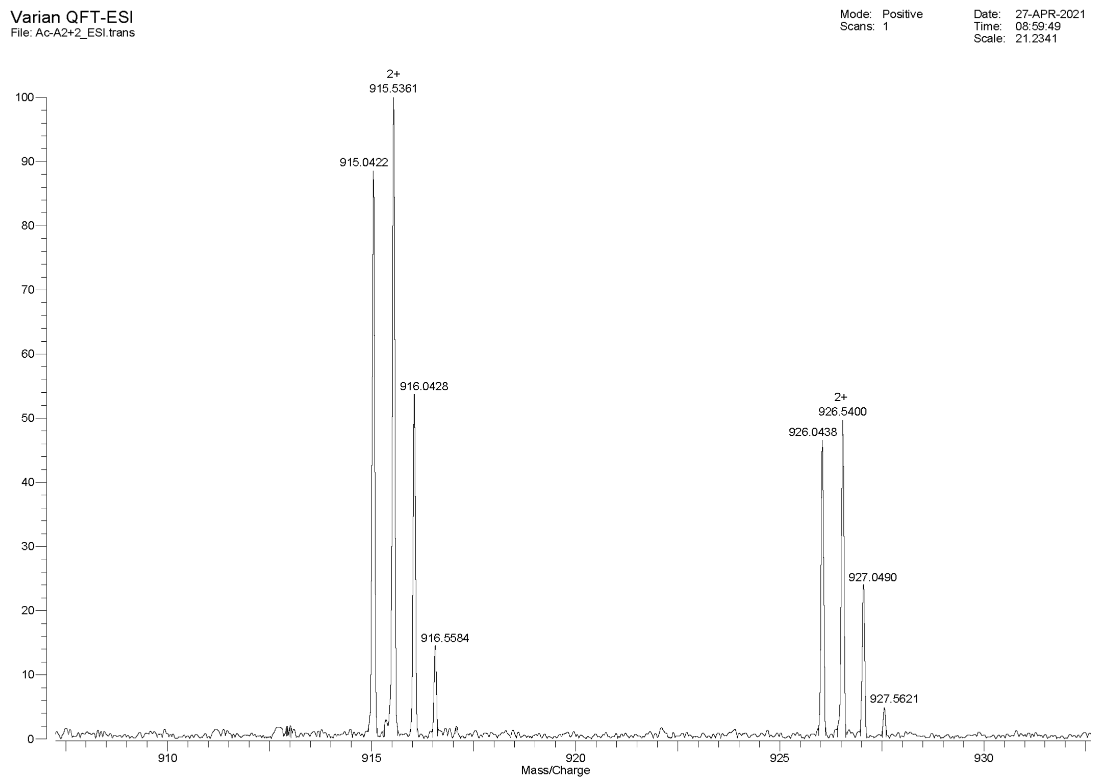

Figure S4. HR-MS spectrum of Ac-GFFY-A2. HR-MS: calc. M = 1829.9739, obsvd. $(M+2H)^{2+}$ = 915.5361.

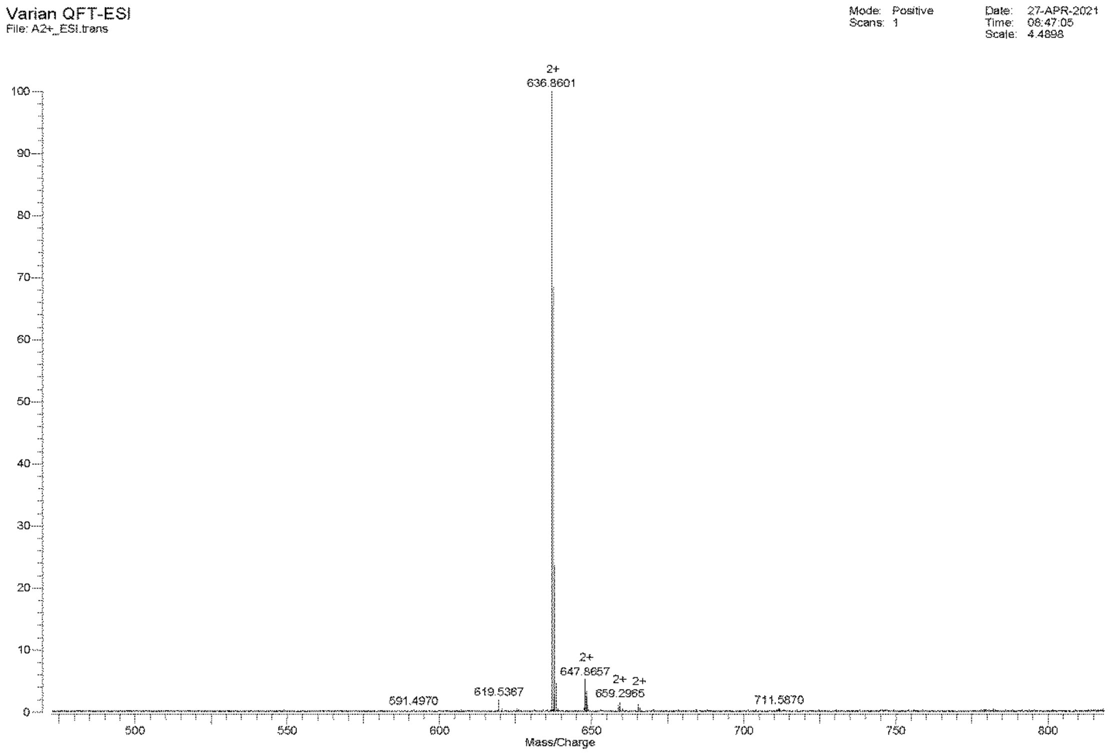

Figure S5. HR-MS spectrum of A2. HR-MS: calc. M = 1272.5180, obsvd. $(M+2H)^{2+}$ = 636.8601.

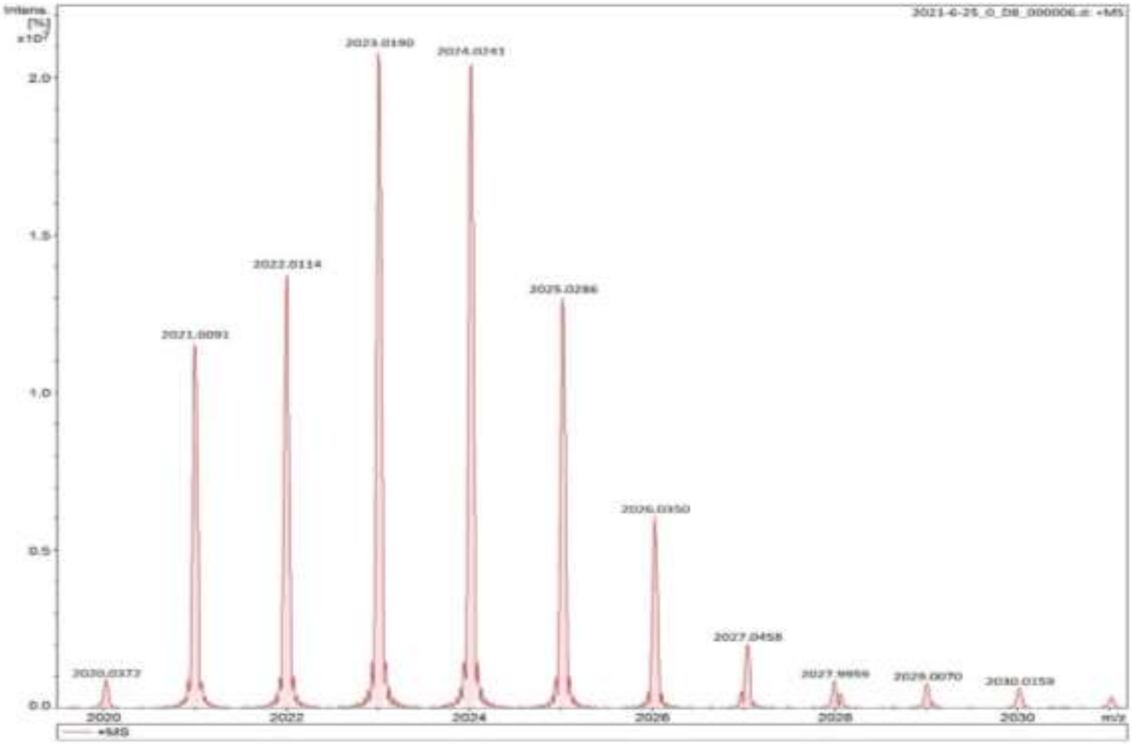

Figure S6. HR-MS spectrum of NBD-GFFY-A2. HR-MS: calc. M = 2021.2710, obsvd. $(M+H)^+$ = 2022.0114.

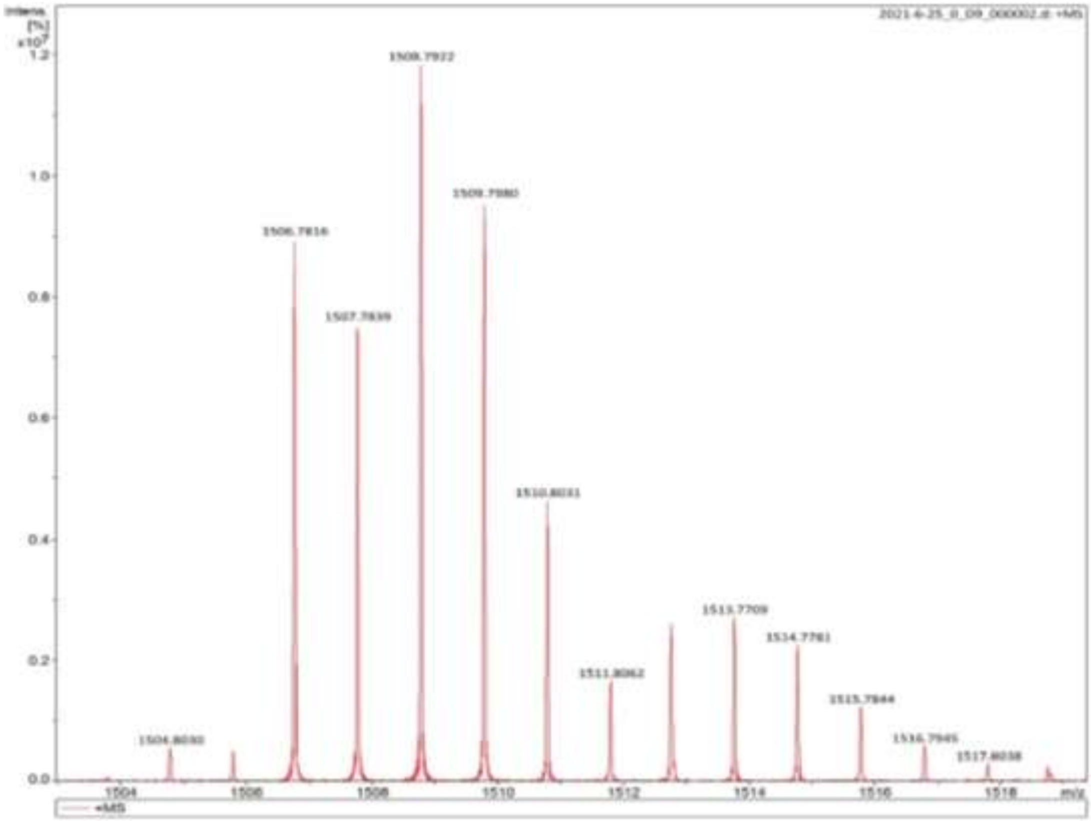

Figure S7. HR-MS spectrum of NBD -A2. HR-MS: calc. M = 1507.7806, obsvd. $(M+H)^+$ =1508.7922.

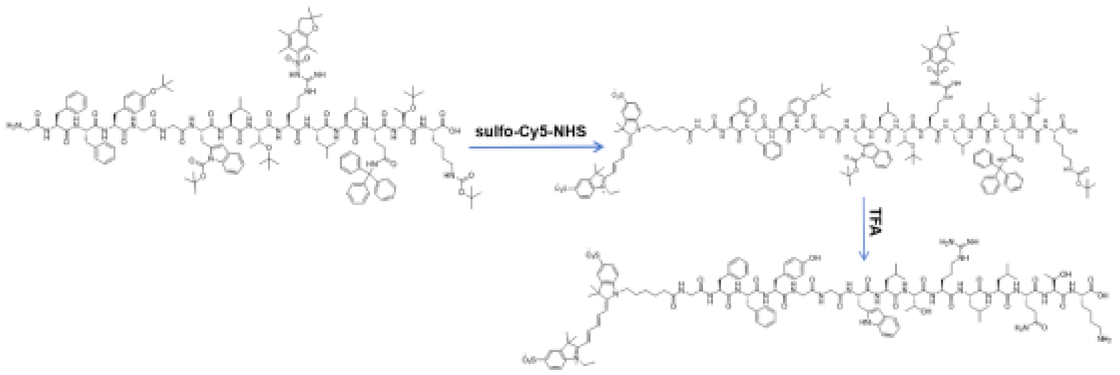

Figure S8. The synthesis procedure of sulfo-Cy5 labeled GFFY-A2 peptide (the sulfo-Cy5 labeled A2 with same procedure)

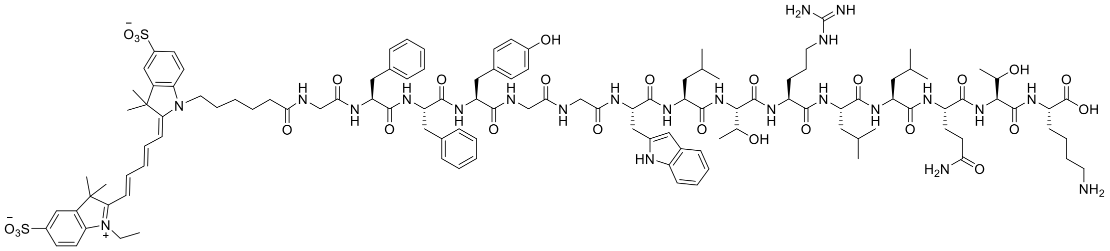

Figure S9. Chemical structure of peptide sulfo-Cy5-GFFY-A2

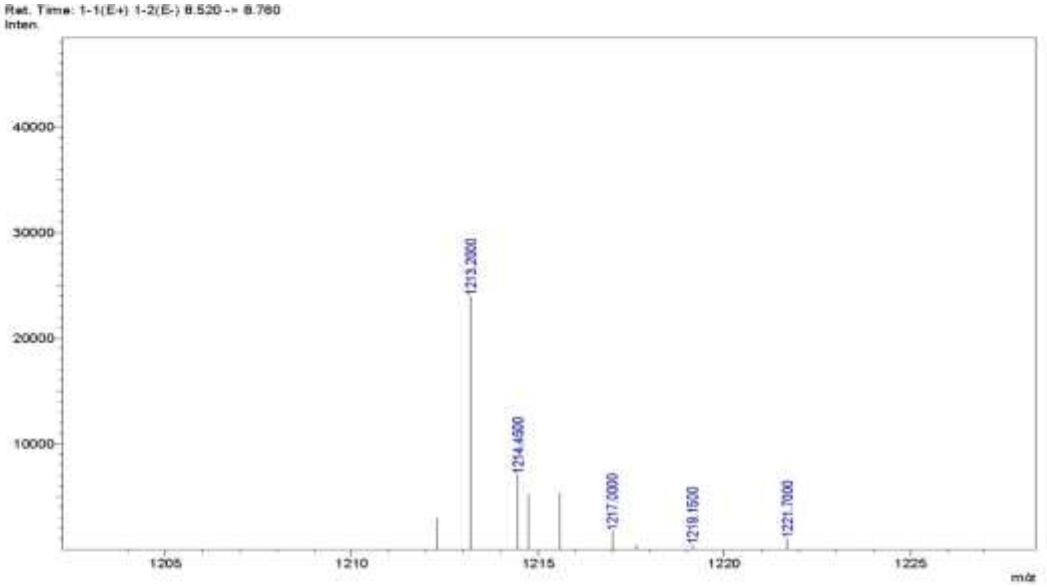

Figure S10. MS spectrum of sulfo-Cy5-GFFY-A2. HR-MS: calc. M = 2424.8865, obsvd. $(M+H)2^{+}$ =1213.20

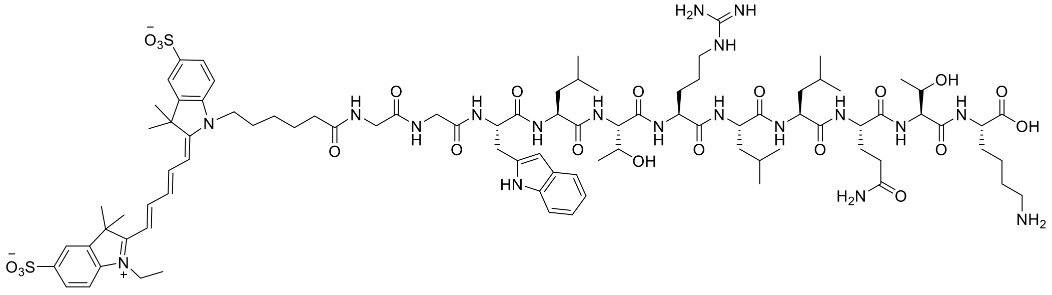

Figure S11. Chemical structure of peptide sulfo-Cy5-A2

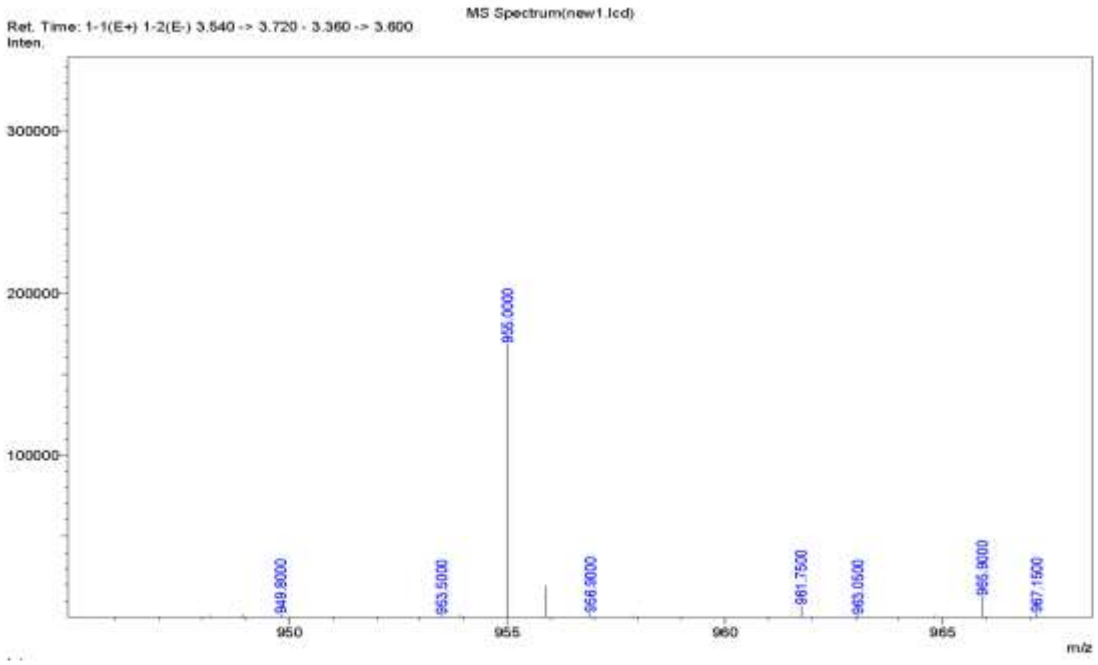

Figure S12. MS spectrum of sulfo-Cy5-A2. HR-MS: calc. M = 1910.3045, obsvd. $(M+H)2^{+}$ =955.00

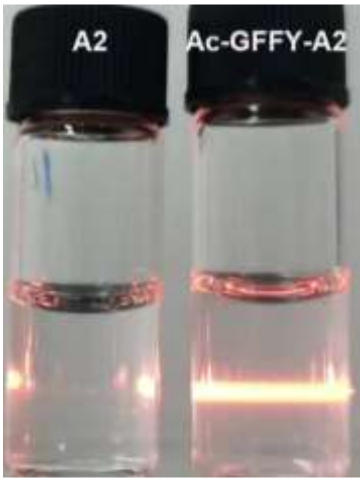

Figure S13. Optical images of tyndall effects of Ac-GFFY-A2 or A2 in saline buffer solution (0.5 wt%).

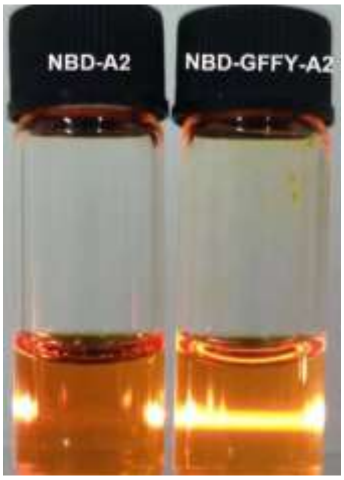

Figure S14. Optical images of tyndall effects of NBD-GFFY-A2 or NBD-A2 in saline buffer solution (0.5 wt%).

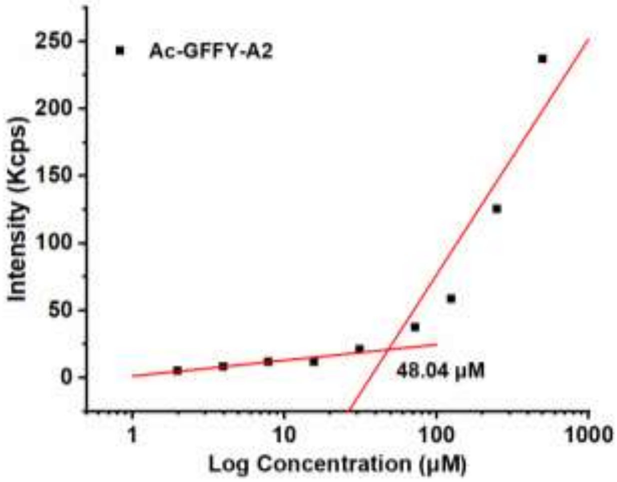

Figure S15. Critical aggregation concentration (CAC) value of Ac-GFFY-A2 sample.

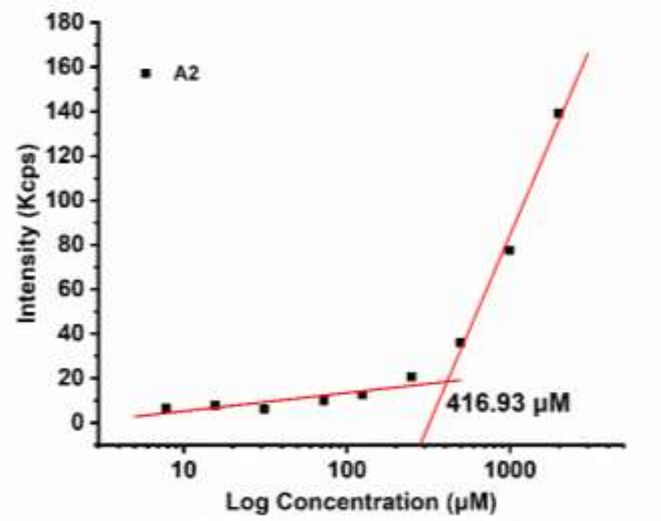

Figure S16. CAC value of A2 sample.

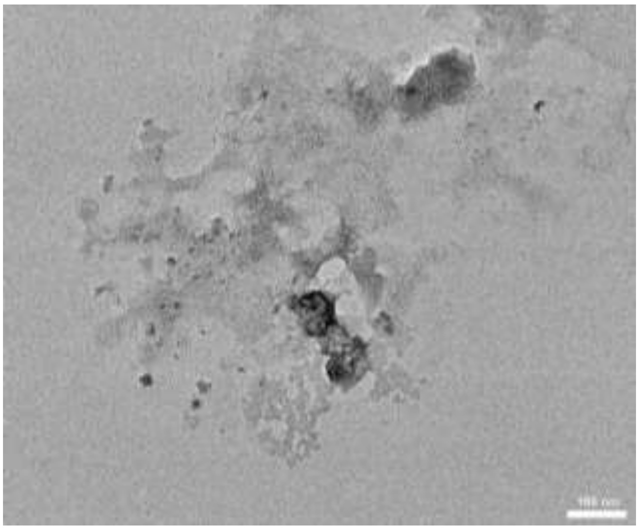

Figure S17. TEM images of A2 in saline buffer at the peptide concentration of 0.5 wt%.

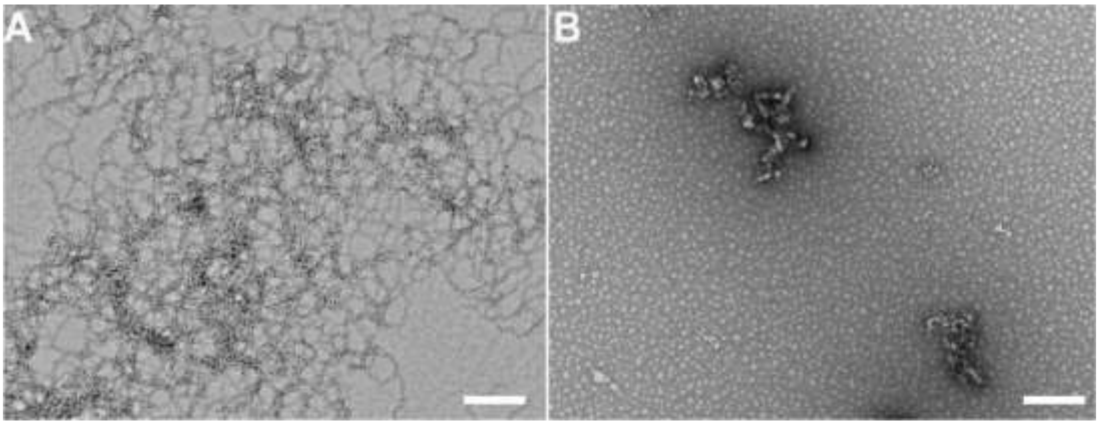

Figure S18. TEM images of A) NBD-GFFY-A2, B) NBD-A2 in saline buffer at the peptide concentration of 0.5 wt%, bar represents 100 nm.

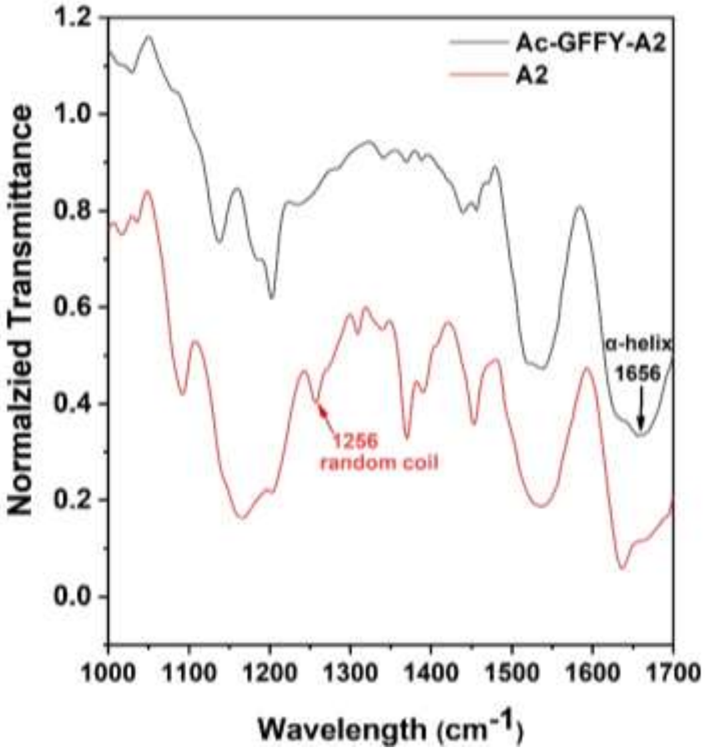

Figure S19. FTIR spectra of Ac-GFFY-A2 and A2 (0.5 wt%) in saline buffer.

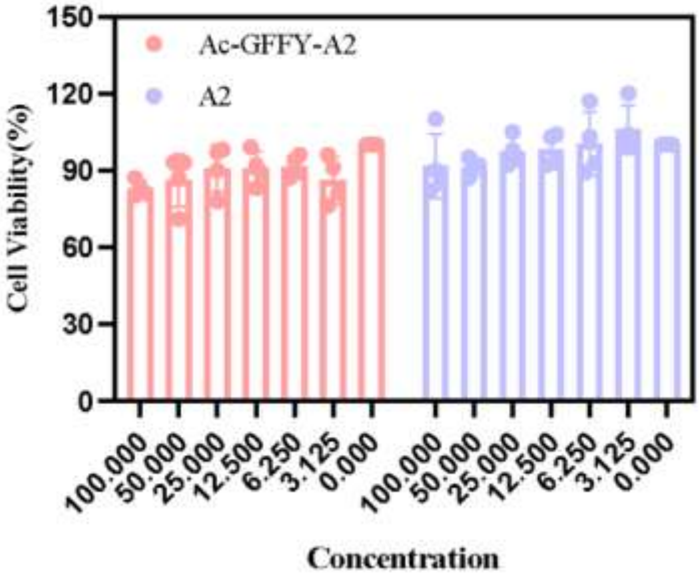

Figure S20. Histogram of the cell viability of MRC-5 cells in the presence of the A2 or Ac-GFFY-A2 (n=4, one-way ANOVA).

TRB3 (green) and p62 (red) in the Figure 3b colocalization analysis by image J software:

According to Figure 3b results, TRB3 can be expressed in the whole MRC-5 cell, while p62 protein is only expressed in the MRC-5 cytoplasm. The difference of

protein expression location, especially the abundant TRB3 in the nucleus, will affect the accuracy of Pearson coefficient. Therefore, we used the Plot Profile method of Image J software to analyze the co-localization of the two proteins. In additional, we also selected regions with more p62 protein for Pearson coefficient calculated by Coloc 2 of Image J.

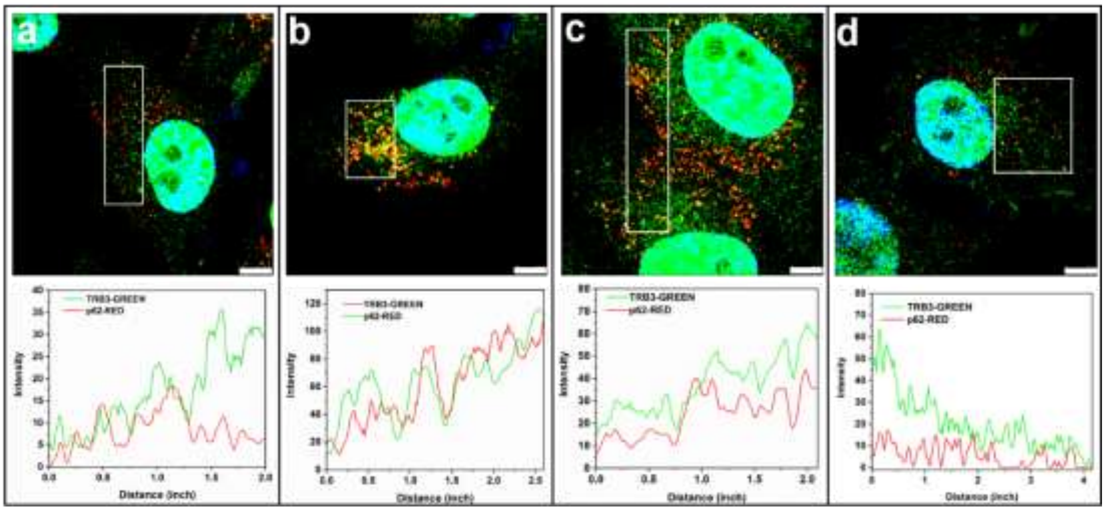

Figure S21. Plots of pixel intensity along the white box from a) Control group, b) TGF-β1+ saline group, c) TGF-β1+ A2, d) TGF-β1+ Ac-GFFY-A2 of images to the top of each plot.

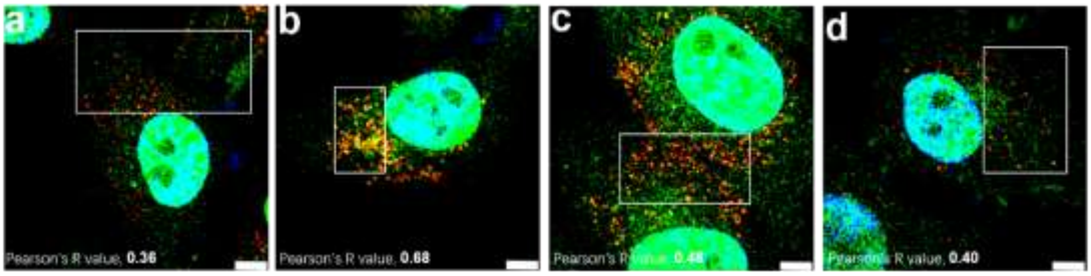

Figure S22. Pearson correlation coefficient in the white box from a) Control group, b) TGF-β1+ saline group, c) TGF-β1+ A2, d) TGF-β1+ Ac-GFFY-A2, calculated by Coloc 2 of Image J.

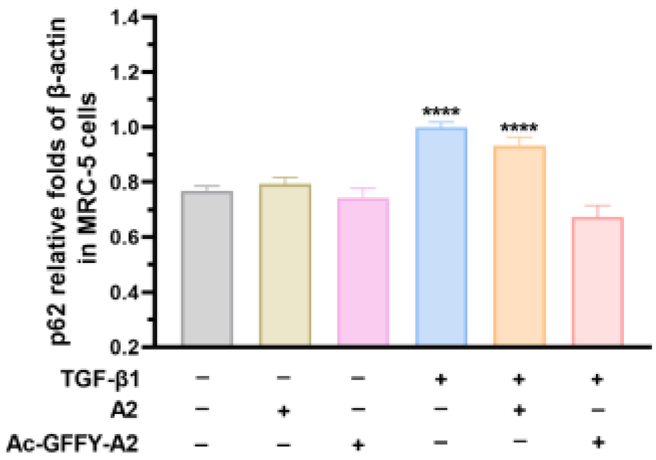

Figure S23. Grayscale analysis of the ratio of p62 to β-actin in MRC-5 cells, (n=3, one-way ANOVA), ****P<0.0001.

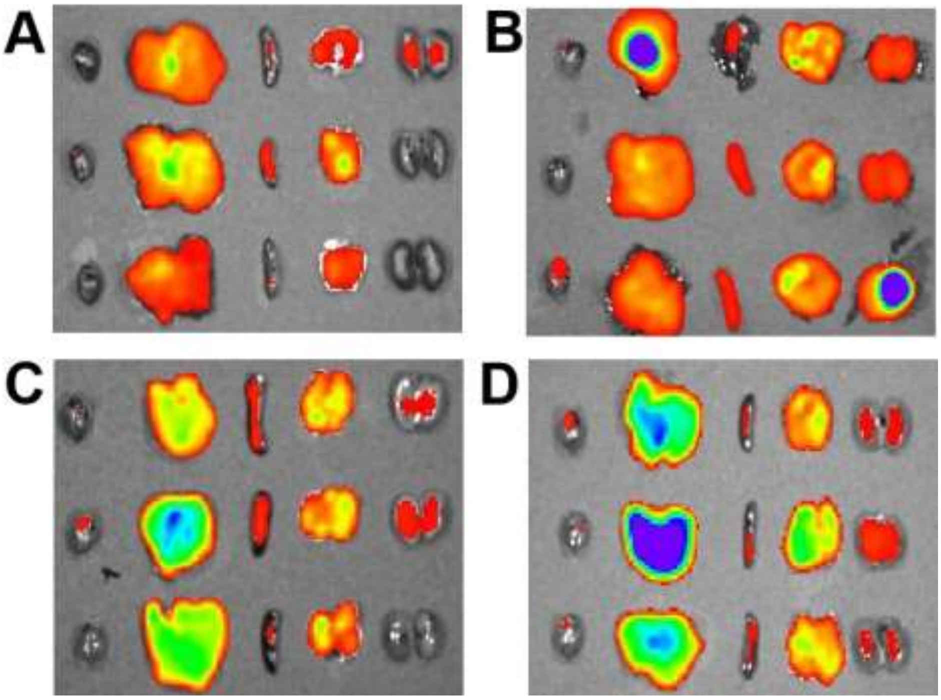

Figure S24. NIR fluorescence images of excised organs of **A**) Cy5-A2-0 day, **B**) Cy5-A2-14 day, **C**) Cy5-GFFY-A2-0 day, **D**) Cy5-GFFY-A2-14 day, post injection at 8 h. From left to right in this picture, the organ was heart, liver, spleen, lung and kidney, respectively.

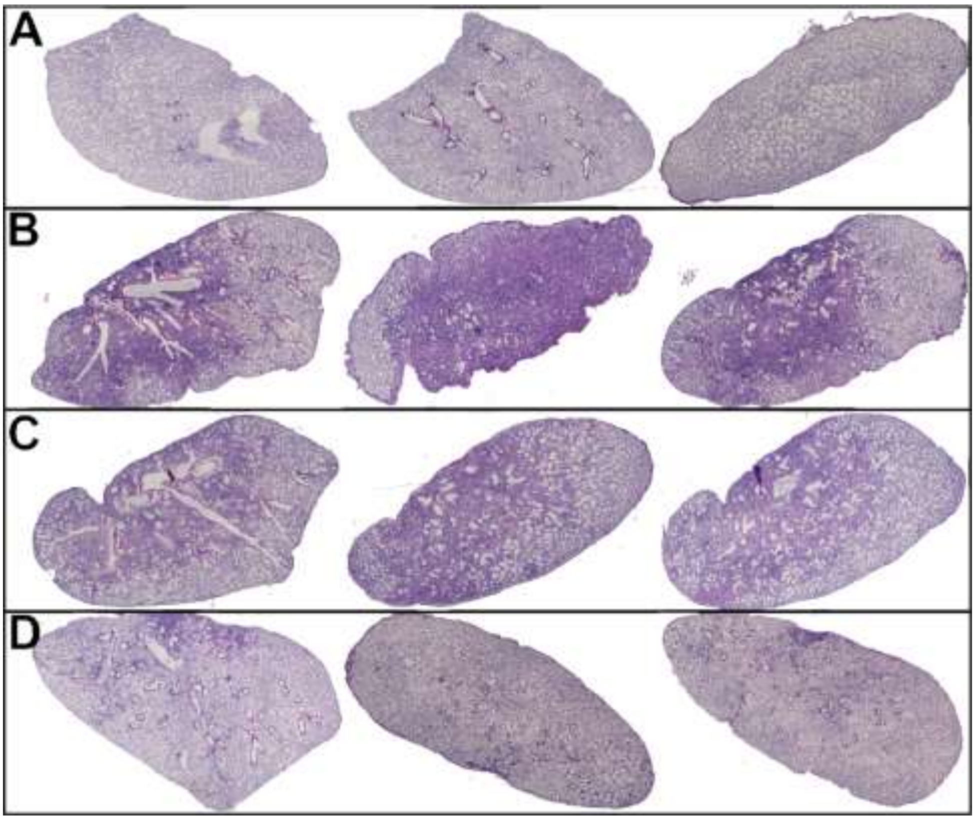

Figure S25. Representative H&E images of whole left lung sections of A) saline + saline, B) BLM + saline, C) BLM + A2, D) BLM+Ac-GFFY-A2.

Methods

Transmission electron microscopy (TEM)

The samples (0.5 wt%, 10 μL) were loaded onto copper mesh coated with carbon. After excess liquid was removed by a filter paper, 2% uranium acetate was used for dyeing the sample for 1 minute and the sample was dried overnight in a dryer. The samples were then imaged at 200 kV using a Tecnai G2 F20 system.

Circular dichroism (CD) spectrum

A BioLogic (MOS-450) system was used to record the circular dichroism spectrum. Test wavelengths was in the range from 180 nm to 280 nm. The samples were added in a 0.1 cm quartz spectrophotometer cell (20-C/Q/0.1), the CD spectrum of saline was used to be background group.

Fourier transform infrared (FTIR) experiment

The Ac-GFFY-A2 (4 mg) or A2 (4 mg) peptide was dissolved in saline buffer (800 μL), respectively. And an appropriate amount of Na_2CO_3 solution (1M) was added to adjust the pH to 7.4. Then the solution was treated by heat-cooling to obtain the peptide assemblies. Before conducting the standard FTIR experiment (BRUKER, TENSOR 37), the peptide assemblies in saline were lyophilized to obtain powder.

Critical aggregate Concentration (CAC)

The CAC values were determined by dynamic light scattering (DLS). Solutions containing different concentration of compound in saline buffer were tested and the light scattering intensity was recorded for each concentration analyzed.

Cell line culture

The human lung fibroblast MRC-5 cells were maintained in Eagle's Minimum Essential Medium (MEM) with 10% fetal bovine serum (FBS), 100 U/mL penicillin and 100 μg/mL streptomycin in 5% CO_2 at 37℃ in a humidified atmosphere.

Stock solution preparation

The Ac-GFFY-A2 (9.15mg) was dissolved in saline buffer (1 mL), and an appropriate amount of Na_2CO_3 solution (1M) was added to adjust the pH to 7.4. Then the solution was treated by heat-cooling to obtain the stock solution of peptide nanofibers.

Cytocompatibility study

We used MRC-5 cells to perform the cytocompatibility experiment for the Ac-GFFY-A2 and A2 peptide. MRC-5 cells were seeded in 96-well plates at a density of 4×10^3 cells per well for 16 hours, followed by culture medium removal and subsequent addition of the culture medium containing Ac-GFFY-A2 or A2 peptide. The initial concentration of all compounds was 100 µM. After 48 hours, 10 µL of the MTT solution (5 mg mL^{-1}) was added to each well and incubated at 37 °C for another 4 hours. The medium was removed, and 100 µL of DMSO was then added to stop the reduction reaction and dissolve the purple formazan formed within the cells. The optical density of the solution was measured at 490 nm using a microplate reader (Bio RADiMark™, USA).

RNA isolation and qRT-PCR analysis

Total RNA was isolated from lung tissues using TRIGene (GenStar, Beijing, China) and then reverse-transcribed by Star ScriptII First-strand cDNA Synthesis Mix (GenStar, Beijing, China) according to the manufacturer's instructions. qRT-PCR was performed using qPCR SYBR Green Master Mix (Yeasen, Shanghai, China). Gene expression was determined relative to that of the endogenous reference gene (Gapdh) using the $2^{-\Delta\Delta Ct}$ method. The mouse primers used in this study were as follows: Acta2 [encoding α-SMA], 5′-GCTGGTGATGATGCTCCCA-3′ and 5′-GCCCATTCCAAC CATTACTCC-3′; Col1a1 [encoding type I collagen (Col1)], 5′-CCAAGAAGACAT CCCTGAAGTCA-3′ and 5′-TGCACGTCATCGCACACA-3′; Fn (encoding fibronectin), 5′-GTGTAGCACAACTTCCAATTACGAA-3′ and 5′-GGAATTTCCG

CCTCGAGTCT-3′; Gapdh (encoding Gapdh), 5′-AGGTCGGTGTGAACGGATTTG -3′ and 5′-TGTAGACCATGTAGTTGAGGTCA-3′.

Live-cell imaging for autophagic flux

According to the manufacturer's instructions, MRC-5 cells were infected with mRFP-GFP-LC3 adenoviral particles. After infection, the cells were cultured for another 24 h and seeded on CLSM dish at a density of 5×10^4 cells. After adherence, the cells were treated with 0 or 10 ng/mL TGF-β1 for 24 h. On the other hand, in order to detect whether peptide could regulate the autophagic flux, the stable infected MRC-5 cells were seeded on CLSM dish at a density of 5×10^4 cells. After adherence, the cells were treated with saline, Ac-GFFY-A2 (50 μM) or A2 (50 μM) plus TGF-β1 (10 ng/mL) for 24 hours. The cells solely treated by saline as control group. Discarding the medium and washing the cells with cold PBS buffer. The autophagic flux was detected by Live Cell Imaging Microscopy (Leica TCS SP5).

Immunofluorescent analysis

The MRC-5 cells were seeded on the CLSM dish at a density of 6×10^4 cells. After adherence, solution containing TGF-β1 (10 ng/mL), saline plus TGF-β1 (10 ng/mL), A2 (50 μM) plus TGF-β1 (10 ng/mL), Ac-GFFY-A2 (50 μM) TGF-β1 (10 ng/mL), was added to incubate cells for 24 h, respectively. Then, the medium was removed and the cells were washed with cold PBS three times. The cells were fixed with 4 % of Paraformaldehyde for 10 min at room temperature. Then, solution was sucked away, and washed by cold PBS three times for a min each time. 0.1% Triton X-100 solution was used to disrupt the cell membrane structure. Then 5 % of goat serum in TBST was used to block non-specific protein binding site and incubate cells for 1 h. Discarding the blocking solution and repeat the washing step mentioned above three times. TRB3 rabbit antibody (1:100) and p62 mouse antibody (1:100) in 5 % goat serum of PBS buffer was used to incubate the cells overnight at 4 ℃. Washing the cells with TBST three times for 10 min each time, the Alexa Fluor®647 goat anti-mouse antibody and Alexa Fluor®488 goat anti-rabbit antibody in 5 % goat

serum of PBS buffer was used to incubate the cells for 1 h at room temperature. Solution of primary antibody was removed and cells were washed three times by PBS buffer and then stained with 0.5 μg/mL of DAPI for 5 min at room temperature. Operations need to be protected from light. All images were taken by a laser scanning confocal microscopy (Leica TSC SP5) at the same voltage.

Cellular uptake

MRC-5 cells were seeded on a 6-well plate at a density of 6×10^4 cells per well then cultured for 12 hours to adhere the walls. The NBD-GFFY-A2 (100 μM) or NBD-A2 (100 μM) was incubated with MRC-5 cells for 4 hours. Discarding the peptide medium and washing the cells with cold PBS three times, then adherent cells were digested into single cell suspensions using trypsin. The cells were collected and centrifuged, finally the cellular uptake was measured by flow cytometry.

Cell migration and invasion assay

For wound healing, cell monolayers were scratched with pipette tip. Cells were incubated with or without 10 ng/ml recombinant human TGF-β1. Ac-GFFY-A2 ((50 μM) or A2 (50 μM) was added to the medium 1 h behind to TGF-β1 stimulation. Images were captured at 0 h and 48 h after scratching, and the lesion area was measured with Image J software. Cell invasion assay was performed using transwell chambers (8-μm pore size, Millipore). Chambers were pre-coated with Matrigel (30 μg per well, BD). Cells were seeded into the chamber with MEM supplemented with 0.1% FBS, and the lower well was added MEM supplemented with 20% FBS. After incubated for 24 h, non-invaded cells on the upside were removed with cotton swap, and invaded cells were fixed with 4% paraformaldehyde, stained with 0.5% crystal violet, and then photographed under a microscope. The crystal violet stain was eluted in alcohol and quantified at OD540 nm. The transwell migration assay procedure was same as invasion assay except that the chamber was not coated with matrixgel.

Western blot analysis and co-immunoprecipitation

Total proteins were exacted from cells or tissues with RIPA lysis buffer (Beyotime Biotecnology). The protein concentration was measured using BCA Protein Assay Kit (Thermo Scientific). Equal amounts of lysates were resolved by SDS–PAGE. The protein transferred PVDF membranes were incubated with following primary antibodies at 4°C overnight: Then the membranes were incubated with horseradish peroxidase (HRP)-conjugated secondary antibodies at room temperature for 1 h, and protein signals were detected using an enhanced chemiluminescence kit (Pierce). For co-immunoprecipitation, exacted proteins were subjected to immunoprecipitation with indicated antibodies overnight at 4℃ with gentle agitation, followed by incubation with Protein A/G-Agarose (GE healthcare) for 4 h at 4℃. The co-precipitates were resolved using SDS–PAGE and detected with immunoblotting.

Bleomycin model of pulmonary fibrosis

Mice were intratracheally administered with bleomycin at a dose of 2 U/kg body weight as previously described.[1]

Living image

The fibrosis mice were randomly after 14 days of bleomycin treatment, and the wild mice were so (0 day). The mice were divided into four groups (n =3), including of Cy5-GFFY-A2 -14 day, Cy5-A2-14 day, Cy5-GFFY-A2-0 day and Cy5-A2-0 day. The mice were then intravenously injected with 200 μL of sulfo-Cy5 labeled peptide (0.3 mg per mouse). Fluorescence imaging was performed using a IVIS Lumina II imaging (PerkinElmer, USA) at different time point post injection. Mice were sacrificed after 8 h post-injection for fluorescence imaging of the major organs (i.e., heart, liver, spleen, lung and kidney).

Hydroxyproline assay

Hydroxyproline content of right lung of each mouse was measured using a conventional hydroxyproline method as previously described.[1]

Histology and immunohistochemistry

The left lungs cleared of blood were inflated with 10% neutral buffered formalin and fixed overnight. Then the tissues were embedded in paraffin and sectioned. The 5-μm sections were stained with H&E and Masson trichrome according to the manufacturer`s instructions (Sigma-Aldrich). Immunohistochemistry was performed as previously described.[2] The antibody specific for α-SMA and Collagen I were used for staining.

Statistical analysis

All results are represented as the means ± SEM. Two group comparisons were analyzed by Student's t-test as appropriate, while multiple comparisons among three or more groups were performed using one-way ANOVA or two-way ANOVA, respectively; $p^* < 0.05$ or $p^{**} < 0.01$, were considered statistically significant. All analyses were performed using GraphPad Prism 8.0 or Origin 2018 software.

Reference:

[1] J. Guo, Y. Fang, F. Jiang, L. Li, H. Zhou, X. Xu, W. Ning, *Eur J Pharmacol* **2019**, *864*, 172712.

[2] S. Gao, X. Li, Q. Jiang, Q. Liang, F. Zhang, S. Li, R. Zhang, J. Luan, J. Zhu, X. Gu, T. Xiao, H. Huang, S. Chen, W. Ning, G. Yang, C. Yang, H. Zhou, *Sci Adv* **2022**, *8* (38), eabo0987.
